# Impact of regulatory variation across human iPSCs and differentiated cells

**DOI:** 10.1101/gr.224436.117

**Published:** 2018-01

**Authors:** Nicholas E. Banovich, Yang I. Li, Anil Raj, Michelle C. Ward, Peyton Greenside, Diego Calderon, Po Yuan Tung, Jonathan E. Burnett, Marsha Myrthil, Samantha M. Thomas, Courtney K. Burrows, Irene Gallego Romero, Bryan J. Pavlovic, Anshul Kundaje, Jonathan K. Pritchard, Yoav Gilad

**Affiliations:** 1 Department of Human Genetics, University of Chicago, Chicago, Illinois 60637, USA;; 2 Department of Genetics, Stanford University, Stanford, California 94305, USA;; 3 Department of Medicine, University of Chicago, Chicago, Illinois 60637, USA;; 4 Department of Biomedical Informatics, Stanford University, Stanford, California 94305, USA;; 5 Department of Biology, Stanford University, Stanford, California 94305, USA;; 6 Howard Hughes Medical Institute, Stanford University, Stanford, California 94305, USA

## Abstract

Induced pluripotent stem cells (iPSCs) are an essential tool for studying cellular differentiation and cell types that are otherwise difficult to access. We investigated the use of iPSCs and iPSC-derived cells to study the impact of genetic variation on gene regulation across different cell types and as models for studies of complex disease. To do so, we established a panel of iPSCs from 58 well-studied Yoruba lymphoblastoid cell lines (LCLs); 14 of these lines were further differentiated into cardiomyocytes. We characterized regulatory variation across individuals and cell types by measuring gene expression levels, chromatin accessibility, and DNA methylation. Our analysis focused on a comparison of inter-individual regulatory variation across cell types. While most cell-type–specific regulatory quantitative trait loci (QTLs) lie in chromatin that is open only in the affected cell types, we found that 20% of cell-type–specific regulatory QTLs are in shared open chromatin. This observation motivated us to develop a deep neural network to predict open chromatin regions from DNA sequence alone. Using this approach, we were able to use the sequences of segregating haplotypes to predict the effects of common SNPs on cell-type–specific chromatin accessibility.

Understanding the genetic underpinnings of complex traits remains a major challenge in human genetics. Genome-wide association studies (GWAS) have provided a wealth of information about the general properties of loci affecting complex traits. Notably, the majority of these loci lie outside of genes and likely act by modifying gene regulation (Li et al. 2016). Unlike genetic variation within coding regions, it is difficult to identify the molecular effects of noncoding variants and, specifically, it is challenging to predict the mechanisms by which noncoding variants act to affect gene regulation. Consequently, a large body of work has been devoted to understanding how genetic variation affects gene regulation (Gibbs et al. 2010; Degner et al. 2012; Gutierrez-Arcelus et al. 2013; Kilpinen et al. 2013; Lappalainen et al. 2013; Banovich et al. 2014; Battle et al. 2014; The GTEx Consortium 2015; Li et al. 2016). These studies have demonstrated that it is possible to connect loci in putative regulatory regions with the specific genes whose regulation they affect. Studies of the genetics of gene regulation have improved our ability to identify putatively causal regulatory variants. In turn, based on functional regulatory inference, we are able to better identify likely disease variants, even when they do not meet genome-wide significance in GWAS studies (Cusanovich et al. 2012).

Thus, a better understanding of the regulatory role of individual genetic variants is critical for our ability to understand complex disease. Yet, recent work suggests that many of these variants have cell-type- or condition-specific effects, which are difficult to characterize (Farh et al. 2015; Finucane et al. 2015). Indeed, to study context-specific effects of genetic variation, researchers are limited to a few commercially available cell lines, easily accessible tissues (e.g., skin and blood) (Gibbs et al. 2010; Degner et al. 2012), and, more recently, frozen post-mortem tissues (The GTEx Consortium 2015). While studies using these resources have provided valuable insight into the genetic architecture of gene regulation, they do not provide a flexible framework to study inter-individual variation in gene regulation in multiple cell types from the same genotype. In particular, many important cell types cannot be obtained from adult post-mortem samples and regardless, post-mortem (typically frozen) samples are unsuited for functional studies and perturbations that require living cells.

Induced pluripotent stem cells (iPSCs) are generated by transforming somatic cells to an embryonic-like state (Takahashi and Yamanaka 2006; Takahashi et al. 2007; Yu et al. 2007) and can be differentiated into a myriad of somatic cell types representing all three germ layers. Importantly, iPSCs can be generated efficiently using a small number of exogenous factors (Takahashi and Yamanaka 2006; Takahashi et al. 2007; Yu et al. 2007), can be cryopreserved, exhibit unlimited self-renewal, and can be used to generate viable somatic cells upon differentiation (Burridge et al. 2016). These properties make iPSCs a valuable cellular model for the study of gene regulation in a controlled setting. Although some debate remains about whether iPSCs are truly equivalent to embryonic stem cells (ESCs), studies have shown, using well-matched lines, that iPSCs are nearly indistinguishable from ESCs in their molecular profiles and their ability to differentiate (D'Aiuto et al. 2014; Pagliuca et al. 2014; Choi et al. 2015; Davidson et al. 2015).

Furthermore, recent work has demonstrated that gene expression and DNA methylation in iPSCs vary significantly and reproducibly among donors (Rouhani et al. 2014; Burrows et al. 2016; DeBoever et al. 2017; Kilpinen et al. 2017), suggesting that iPSCs can be used to study the impact of genetic variants on gene regulation. Indeed, genetic variation appears to be the main driver of gene expression variation in iPSCs (Kilpinen et al. 2013; DeBoever et al. 2017), an observation that is robust with respect to a large number of technical considerations, including the somatic cell type from which the iPSC was generated. Thus, once differentiated into relevant cell types, iPSC-derived cells can be used to study the regulatory effects of disease-associated variants.

Here, we report the reprogramming of 58 Yoruba lymphoblastoid cell lines (LCLs) into iPSCs, of which 14 were further differentiated into cardiomyocytes. Previously, our group extensively studied gene regulatory variation in the Yoruba LCLs. The establishment of iPSCs from a panel of well-studied individuals allowed us to track the effects of genetic variation on gene regulation following cell reprogramming and differentiation. We therefore explored the utility of iPSCs and iPSC-derived cells to study the impact of genetic variation on gene regulation in multiple cell types. In particular, measuring DNA methylation, chromatin accessibility, and RNA expression levels in multiple individuals and multiple cell types allowed us to study the mechanisms by which genetic variation affects gene regulation in a cell-type–specific manner.

## Results

### Generation of a panel of iPSCs from 58 Yoruba individuals

We generated a panel of iPSCs from 58 well-characterized Yoruba LCLs. Briefly, LCLs were reprogrammed using a previously-described episomal approach (Okita et al. 2011). After a week in suspension, cultured cells were seeded onto a layer of gelatin and mouse embryonic fibroblasts. A single colony was obtained from each line and passaged for 10 wk before final characterization, conversion to feeder-free growth, and collection. Pluripotency and stability were confirmed for each line (Supplemental Fig. S1; Supplemental Materials). This panel represents the largest stock of characterized nonEuropean iPSCs to date and is available to other researchers, complementing parallel efforts in Europeans (see Data Accession in Supplemental Materials; Kilpinen et al. 2017).

**Figure 1.**
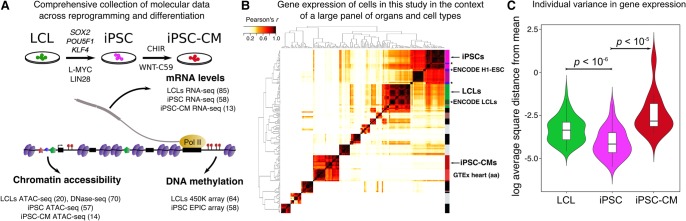
Systematic measurements of molecular phenotypes across reprogramming and differentiation. (*A*) Summary of data collection. (*B*) Correlation matrix of gene expression from our samples and samples from ENCODE (*) and GTEx. Our LCL samples cluster most closely with LCLs samples from ENCODE, while our iPSCs and iPSC-CM lines cluster most closely with H1-ESC (ENCODE) and heart (GTEx), respectively. Dark purple: GTEx bone marrow. (*C*) Violin plots representing per individual log_2_ of the average square distance from the mean (Supplemental Materials) for iPSC, LCL, and iPSC-CM gene expression levels. Plots for chromatin accessibility and DNA methylation levels are shown in Supplemental Figure S7.

To study gene regulation in iPSCs, we assayed three molecular phenotypes: mRNA expression (using RNA-seq; *n* = 58), chromatin accessibility (ATAC-seq; *n* = 57), and DNA methylation levels (EPIC arrays; *n* = 58). We also differentiated 14 iPSC lines into iPSC-derived cardiomyocytes (iPSC-CMs) (Supplemental Materials; Supplemental Table S1) and collected RNA-seq and ATAC-seq from the 14 iPSC-CMs (Fig. 1A). We analyzed these newly collected data jointly with data previously collected from the same Yoruba LCLs (we complemented the original DNase I hypersensitivity data with new ATAC-seq data for 20 of the LCLs). These data were processed using canonical pipelines and procedures (Supplemental Materials; Supplemental Figs. S2–S6).

Given the in vitro nature of the cell types reported here, we sought to evaluate the similarity of the gene expression patterns with respect to data from a broad panel of primary tissues and other cell types. Using RNA-seq data from a panel of tissues and cell types from GTEx (The GTEx Consortium 2013) and ENCODE (The ENCODE Project Consortium 2012), respectively, gene expression data from our LCLs cluster most closely with data from ENCODE LCLs, as expected. Similarly, gene expression data from our iPSCs cluster with data from H1 embryonic stem cell lines from ENCODE, and data from our iPSC-CMs cluster most closely with gene expression data from GTEx heart tissues (atrial appendages) (Fig. 1B; Supplemental Materials). Thus, our cultured cells broadly recapitulate expected regulatory patterns.

### Regulatory variation in three different cell types

We compared molecular data across the three cell types using the log_2_ average square distance from the mean (Supplemental Materials); we observed that chromatin accessibility, gene expression, and DNA methylation levels were all more homogenous between individuals in iPSCs than in LCLs or iPSC-CMs (*P* < 10^−5^ for all comparisons) (Fig. 1C; Supplemental Fig. S7). Furthermore, a similar increase in expression variability is observed in primary heart tissue (Supplemental Materials). This is consistent with the notion that developmental processes are canalized (Waddington 1959) and that regulatory states in embryonic cells are tightly controlled.

After examining overall properties in our data, we sought to characterize the effect of genetic variation on gene regulation. While there have been numerous multitissue studies of expression and expression quantitative trait loci (eQTLs), there is a paucity of our data on QTLs for chromatin accessibility (caQTLs) outside of LCLs (Cheng et al. 2016; Alasoo et al. 2017), and this study represents the first characterization of caQTLs within iPSCs in combination with an iPSC-derived cell type.

We first analyzed data from each cell type independently. We identified thousands of putatively *cis* genetic associations with all three regulatory phenotypes at 10% FDR (Supplemental Materials; Supplemental Table S3). Despite the observation that regulatory phenotypes are associated with lower inter-individual variation in iPSCs compared to LCLs, we found similar or greater numbers of expression QTLs in iPSCs when sample sizes are matched across cell types (e.g., 1441 eQTLs in iPSCs versus 1168 in LCLs using 58 individuals). In addition, using WASP, a powerful approach that leverages allelic imbalance measurements to identify molecular QTLs when sample sizes are small (van de Geijn et al. 2015), we identified 517 eQTLs and 4045 chromatin accessibility QTLs in differentiated iPSC-CMs (14 individuals). In general, we observed a high degree of QTL sharing between cell types. We found 71% to 91% overlap (depending on our choice of *P*-value cutoff in the eQTL discovery cell type) in eQTLs between iPSCs and LCLs, using an estimate of sharing that accounts for incomplete power of the replication tests (Storey's π_0_) (Supplemental Fig. S9). The proportion of sharing is lower when considering iPSC-CMs (Supplemental Fig. S9), as expected given the difference in sample size.

### Cell-type–specific open chromatin explains cell-type–specific QTLs

**Figure 2.**
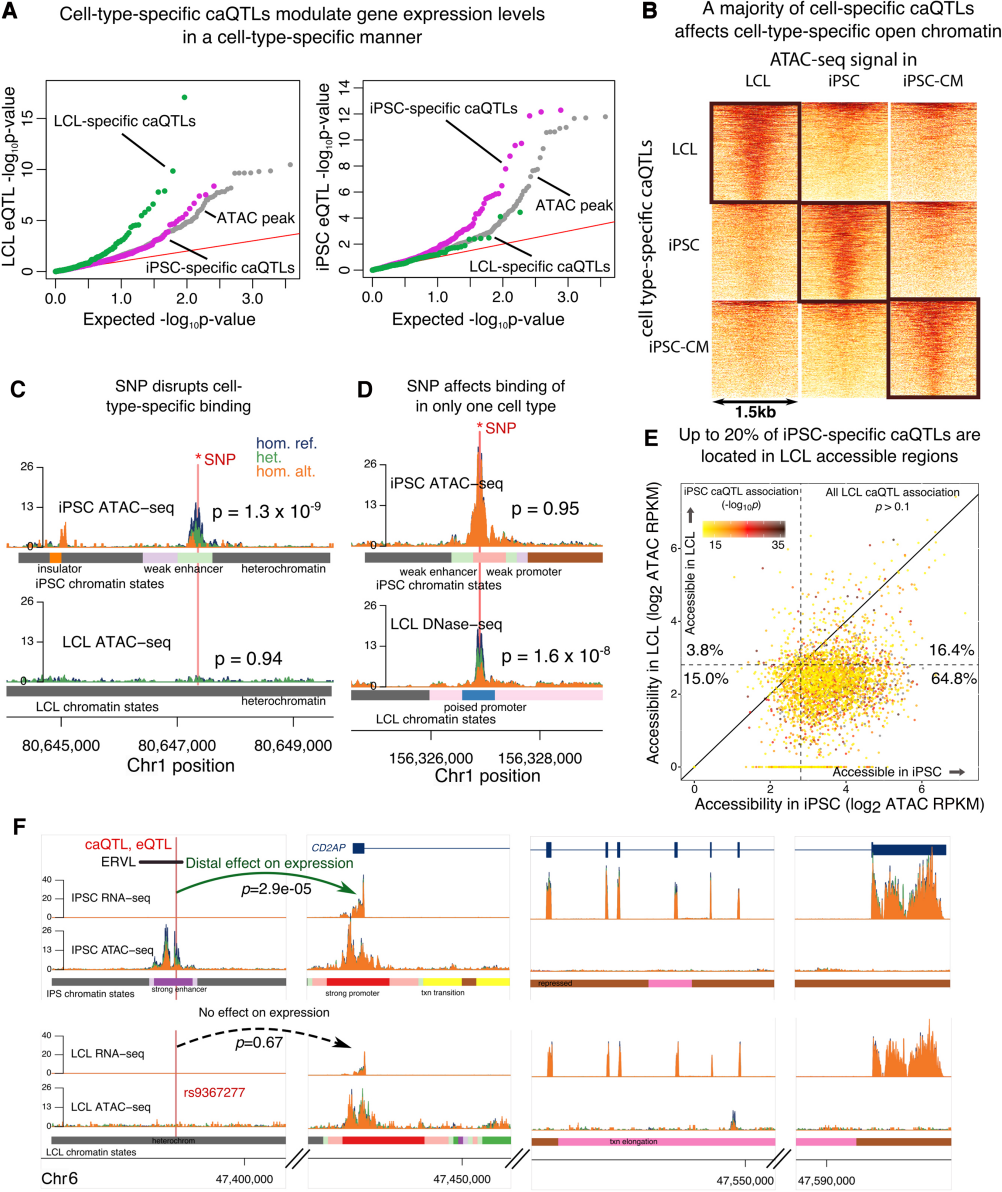
Mechanisms of cell-type–specific regulatory variation. (*A*) QQ-plot of LCL and iPSC eQTL signal conditioned on LCL- and iPSC-specific caQTLs. Higher enrichment of LCL (iPSC) eQTLs among LCL (iPSC) caQTLs links cell-type–specific regulation of chromatin accessibility to cell-type–specific regulation of gene expression. (*B*) Chromatin accessibility signal around cell-specific caQTLs in corresponding cell types (black rectangles) and in other cell types. A lack of accessibility in other cell types suggests that cell-specific caQTLs often affect cell-specific accessible regions, e.g., *C*. (*C*,*D*) Examples of cell-type–specific regulatory effects of genetic variation. SNP is correlated with accessibility of an iPSC-specific open chromatin region in iPSCs only (*C*) or of a nonspecific open chromatin region in LCLs only (*D*). (*E*) Scatter plot of iPSC and LCL chromatin accessibility at iPSC-specific caQTLs. About 20% of iPSC-specific caQTLs are accessible in LCLs. Plot of LCL-specific caQTLs in Supplemental Figure S15. (*F*) Example of an iPSC-specific caQTL that is also an iPSC-specific eQTL. SNP rs9367277 is associated with both chromatin accessibility of a strong enhancer and with expression of the *CD2AP* gene in iPSCs. Interestingly, rs9367277 lies in a transposable element of the ERVL family, which is preferentially activated in embryonic stem cells (Kunarso et al. 2010).

The high sharing of regulatory QTLs across cell types notwithstanding, we asked about the mechanisms by which a subset of genetic variants affects gene regulation in one cell type with no detectable effect in other cell types. Such a pattern is of particular interest given that disease-associated variants are enriched in cell-type–specific open chromatin (Finucane et al. 2015). We thus wondered whether genetic variants in cell-type–specific open chromatin often drive cell-type–specific variation in gene regulation. In LCLs, about 2/3 of eQTLs are due to variants that alter chromatin accessibility or histone marking (Li et al. 2016). Consistent with the idea that cell-type–specific effects at the chromatin level percolate to cell-type–specific gene expression, we found that the iPSC-specific caQTL SNPs we identified (Supplemental Materials) were more likely to affect gene expression levels in iPSCs than were LCL-specific caQTL SNPs and that the converse was also true (*P* = 0.01, *P* = 4.7 × 10^−5^, respectively; Fisher's exact test) (Fig. 2A; Supplemental Tables S4, S5). For over 80% of stringent caQTL-eQTL pairs (Supplemental Materials), we found that the direction of caQTL effects were concordant with that of the associated eQTL (Supplemental Fig. S10). We also found that the magnitudes of caQTL effects were not predictive of the corresponding eQTL effect sizes (Supplemental Fig. S11). However, eQTLs associated with chromatin changes do tend to have larger effect sizes on average (Supplemental Fig. S12).

We further asked about the mechanisms by which genetic variants affect chromatin accessibility broadly, in multiple cell types, or specifically in a single cell type. As expected, caQTLs that are shared across cell types lie within regulatory regions that are accessible in all cell types and likely affect the DNA binding of the same factors (Supplemental Figs. S13, S14). In contrast, most cell-type–specific caQTLs lie in regions that are accessible in the affected cell type but show little or no accessibility in the other cell types (Fig. 2B,C). While this is largely expected, we were able to estimate that >70% of cell-type–specific caQTLs could be explained simply by cell-type–specific regulatory activity (Fig. 2B). In contrast, only 48% of iPSC-specific eQTLs were driven by iPSC-specific activity. Many of these cell-type-specific caQTLs are located quite far from the gene they regulate (e.g., 50 kb or more), and likely function by affecting distal enhancer or promoter elements (Supplemental Fig. S15; Supplemental Table S6). Interestingly, we note that in iPSCs the frequent cell-type–specific activation of enhancers located in the ERV family of transposable elements, consistent with previous work in embryonic stem cells (Fig. 2F; Kunarso et al. 2010), may allow for cell-type–specific evolution of the regulatory network by co-option of the transposed elements as regulatory elements, followed by fine-tuning through the selection of DNA mutations (Kunarso et al. 2010).

While the notion that cell-type–specific caQTLs can often be explained by cell-type–specific chromatin activity is quite intuitive, we also found numerous regions that were accessible in multiple cell types but with a regulatory effect in a single cell type only (Fig. 2D,F; Supplemental Table S6). In fact, up to 20% of cell-type–specific caQTLs are accessible in multiple cell types (Fig. 2E). This observation is consistent with the idea that multiple DNA-binding factors may affect chromatin activity at the same locus by binding to distinct but nearby motifs (Farley et al. 2015; Maurano et al. 2015).

### Sequence-based model for chromatin activity explains the regulatory effects of QTLs

**Figure 3.**
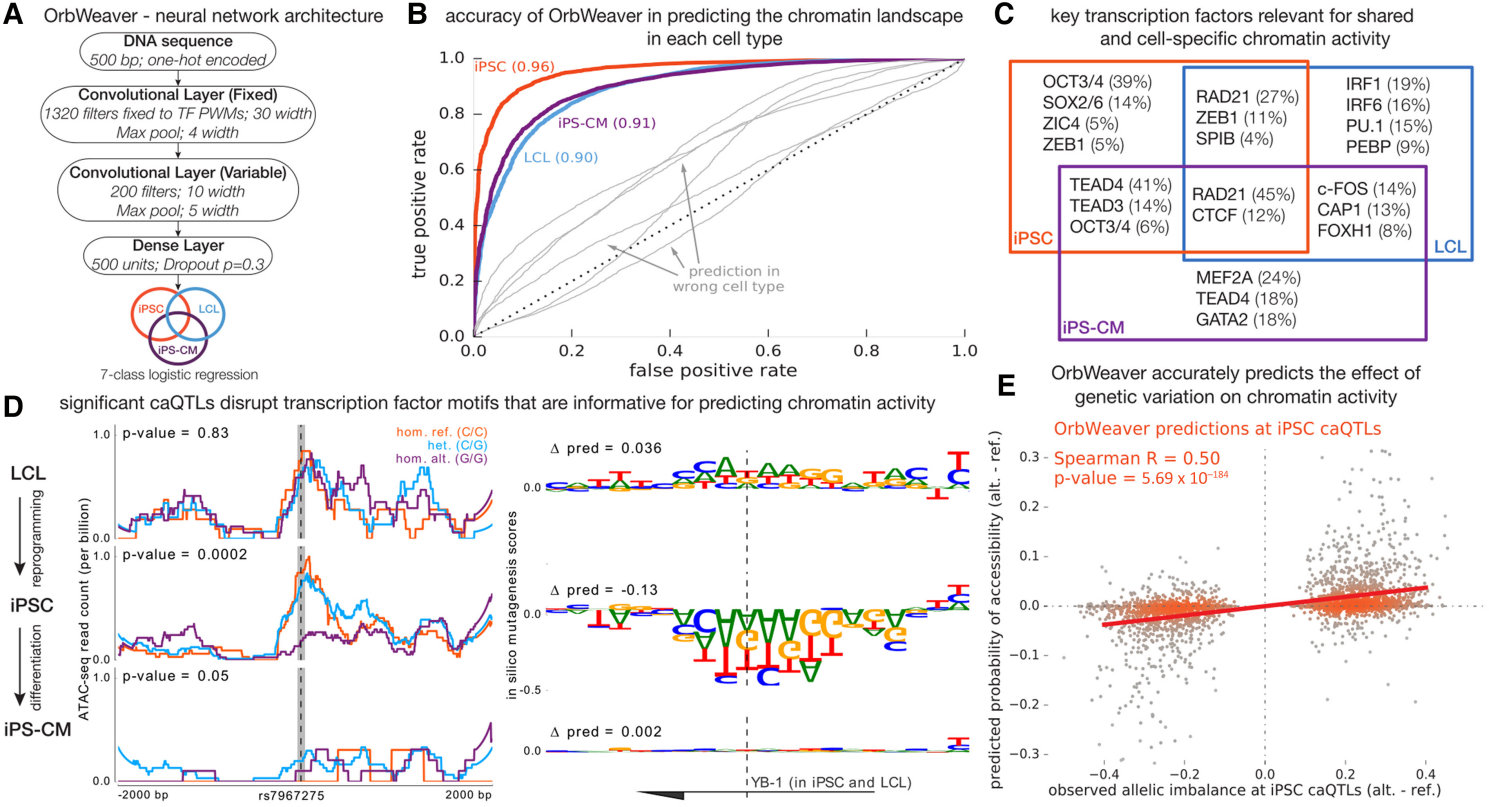
Predicting chromatin activity from sequence using deep neural networks. (*A*) OrbWeaver is a four-layered neural network where the parameters of the first convolutional layer are fixed to known position weight matrices of human transcription factors. The activation function used in each of the convolutional and dense layers is the Rectified Linear Unit (ReLU). (*B*) The OrbWeaver model for one cell type poorly predicts open chromatin in other cell types (gray), highlighting that the model captures cell-type–specific regulatory elements. (*C*) Transcription factors important for each locus were identified using DeepLIFT scores; this panel illustrates the top key TFs for each of the seven categories of chromatin activity and the fraction of loci explained by them. (*D*) An example of a locus that is open in iPSCs and LCLs but was identified to be an iPSC-specific caQTL. The subpanels on the *left* show the raw ATAC-seq signal in each cell type stratified by genotype of the most significant SNP of the iPSC caQTL. The subpanels on the *right* show the marginal change in OrbWeaver predictions due to mutating the reference base at each position to an alternate base. The sequence shown corresponds to the shaded portion on the *left* subpanels, and the reported Δpred values correspond to the change between alleles of the most significant SNP. The TF important for this locus as identified by DeepLIFT is YB-1, a factor highly expressed in all three cell types. (*E*) Scatter plot comparing the observed allelic imbalance at iPSC caQTLs, estimated by WASP, and the predicted difference in median chromatin activity between haplotypes tagged by the two alleles of the causal SNP. Note that the OrbWeaver model was learned using the reference genome sequence alone and had no information regarding genetic variation in the population when learning the model parameters.

Our observations that cell-type–specific open chromatin regions can often explain contrasting effects of genetic variants in different cell types motivated us to explore the sequence features underlying differences in chromatin activity across cell types. In particular, we aimed to identify DNA sequences that could predict cell-type–specific effects of regulatory variants. We investigated the use of machine learning models to predict the chromatin activity of regulatory elements across our three cell types using DNA sequence only (Zhou and Troyanskaya 2015; Hashimoto et al. 2016; Kelley et al. 2016; Zeng et al. 2016). We developed a four-layered neural network architecture, OrbWeaver, to predict cell-type–specific chromatin accessibility of 500-bp windows centered at a regulatory locus (Fig. 3A; Supplemental Fig. S16). In contrast to popular approaches that learn all the parameters of the neural network de novo, we used log-transformed position weight matrices (PWMs) of 1320 human transcription factors (Supplemental Materials; Matys et al. 2006; Jolma et al. 2013) as the first layer of OrbWeaver. As training input, we used 282,088 loci that were identified as accessible in at least one of the three cell types. When testing our predictions on a held-out data set of 7151 loci, we achieved high accuracies in all three cell types: iPSC (AUC = 0.96), LCL (AUC = 0.90), and iPSC-CM (AUC = 0.91) (Fig. 3B; see Supplemental Fig. S17 for precision recall results). We found that the use of transcription factor PWMs as the first layer of OrbWeaver yielded higher predictive accuracies with a simpler neural network architecture than with a more complex architecture that did not use transcription factor PWMs (Supplemental Fig. S17).

To identify transcription factors that help predict the shared and cell-type–specific regulatory activity across loci, we computed DeepLIFT scores (Shrikumar et al. 2016) with respect to each filter in the first convolutional layer. Among 1320 factors for which we had PWMs, the factor with the highest score for a given locus was assigned to be the most important factor for explaining the chromatin activity of said locus. Aggregating the key factor across all loci, we recovered transcription factors that are known to drive cell-type–specific chromatin activity (Fig. 3C) and identified several additional factors that are putatively important for cell-type–specific gene regulation (Supplemental Table S7). Notably, nearly 40% of iPSC-specific open chromatin loci could be explained by the *POU5F1* motif alone. In LCLs and iPSC-CMs, a larger number of TFs are needed to explain the same fraction of cell-type–specific open chromatin loci. This observation is consistent with the higher predictive accuracy achieved for iPSCs compared to LCLs and iPSC-CMs, even with simpler neural network models (Supplemental Fig. S17), and suggests that fewer *trans*-acting factors establish the chromatin landscape in pluripotent cells than in somatic cells.

Given our ability to predict cell-type–specific chromatin activity on a genome-wide scale, from DNA sequence alone, we reasoned that OrbWeaver might also allow us to predict cell-type–specific effects of SNPs on chromatin activity (Fig. 3D). Prediction of SNP effects on gene regulation, especially in specific cell types, is a challenging problem but is an essential task for future interpretation of personal genomes. Starting with iPSC caQTLs, we found that OrbWeaver predictions track the observed allelic imbalance ratio with a correlation of 0.50 (*P* = 6 × 10^−184^) (Fig. 3E). Considering all tested SNPs in open chromatin peaks (the majority of which presumably have no true effect on chromatin accessibility), the correlation is more modest, though highly significant (iPSC correlation 0.12; *P* < 10^−308^). Notably, our ability to predict caQTL effects in one cell type is drastically reduced when using our model for another cell type (Supplemental Fig. S18), indicating that our model has high cell-type specificity. Altogether these findings demonstrate our ability to identify *trans*-acting elements driving cellular differences in chromatin accessibility and, more importantly, to predict effects of genetic variation in a cell-type–specific manner.

### iPSC-differentiated cells capture effects of disease variants

**Figure 4.**
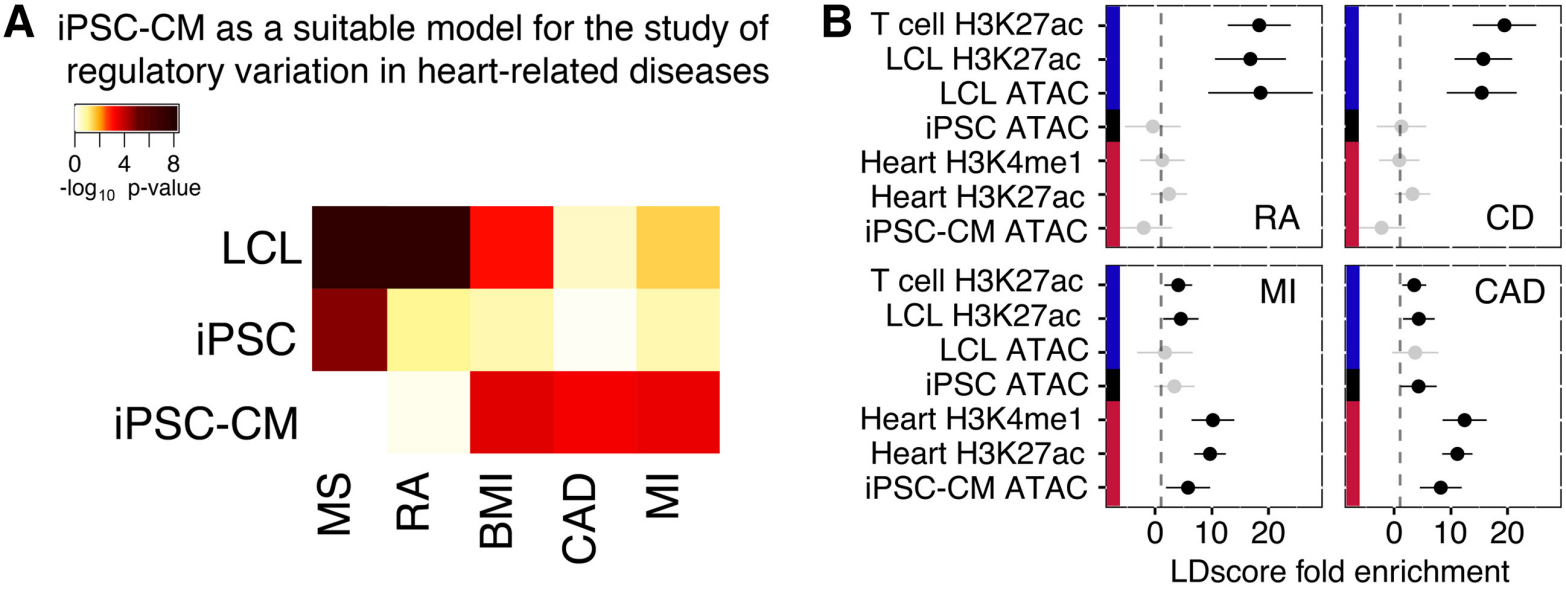
Modeling complex disease using iPSC-derived cells. (*A*) Heat map of enrichment *P*-values of GWAS signals near genes with cell-type–specific expression (Supplemental Materials). (*B*) Enrichments of SNPs associated with four different diseases in different partitions of the genome (computed using LDscore regression; point estimates ±95% confidence intervals). In both analyses, the autoimmune traits (multiple sclerosis [MS] or Crohn's disease [CD] and rheumatoid arthritis [RA]) show enrichment near genes and chromatin that are more active in LCLs, and the heart-related traits (coronary artery disease [CAD] and myocardial infarction [MI]) are enriched in iPSC-CM active regions.

Ultimately, the iPSCs and their derived cell types may be valuable for developing a variety of models of human disease, provided that cultured differentiated cells are an effective system with which to model gene regulation in the corresponding primary tissue. We evaluated the fidelity of iPSC-CMs as a model for heart tissues and heart-related diseases. As discussed above, gene expression from iPSC-CMs most closely resembles that of GTEx heart samples. Furthermore, eQTLs detected in our iPSC-CMs are most enriched for eQTLs identified in GTEx heart tissues (left ventricle) (Supplemental Fig. S8). We used a polygenic method (Supplemental Materials) to identify enrichments of GWAS signals associated with genes whose expression shows cell-type specificity. Genes more specifically expressed in iPSC-CMs are enriched for signals from GWAS for body mass index (BMI), coronary artery disease (CAD), and myocardial infarction (MI), while genes more specifically expressed in LCLs are enriched for signals from GWAS for multiple sclerosis (MS), and rheumatoid arthritis (RA) (Fig. 4A).

We also used stratified linkage disequilibrium (LD) score regression (Finucane et al. 2015) to estimate enrichment of heritability explained by GWAS signal within open chromatin in the different cell types (Fig. 4B). As expected, heritability explained by SNPs in LCL ATAC-seq peaks were enriched in both autoimmune diseases we tested: Crohn's disease (CD, 15.4-fold, *P* = 2 × 10^−5^) and rheumatoid arthritis (RA, 18.6-fold, *P* = 7 × 10^−5^). For the two heart-related GWAS tested, CAD and MI, we observed a significant enrichment among SNPs in iPSC-CM ATAC-seq peaks (CAD, 8.2-fold, *P* = 2 × 10^−4^; MI, 5.8-fold, *P* = 0.02) and among SNPs in heart H3K27ac peaks (CAD, 11.1-fold, *P* = 4 × 10^−11^; MI, 9.7-fold, *P* = 3 × 10^−9^). However, SNPs in LCL or iPSC ATAC-peaks showed weaker enrichment for CAD (*P* = 0.19 and *P* = 0.05, respectively) and MI (*P* = 0.79 and *P* = 0.20, respectively). The variability in heritability explained by regulatory marks in different cell types suggests that we must be careful in how we assess the suitability of a cell type to model specific diseases. Nevertheless, our observations support the general belief that cellular reprogramming followed by differentiation is a promising strategy to generate disease models for which primary tissue or cell type is difficult to obtain.

## Discussion

We established a unique resource of 58 fully characterized iPSC lines. These lines were reprogrammed from LCLs obtained from Yoruba individuals originally collected as part of the HapMap project. At this time, ours is the largest panel of iPSCs from individuals of African ancestry, and it is available to any interested researcher with no restriction or limitation. Our study design allowed us to characterize multiple regulatory phenotypes (gene expression, chromatin accessibility, and DNA methylation) across three cell types from the same panel of individuals. Using these data, we studied regulatory variation between individuals across cell types at multiple levels. We found that regulatory variation between individuals was lower in iPSCs than in LCLs, cardiomyocytes, and heart tissue. Interestingly, this reduced variation in regulatory phenotypes did not diminish our ability to identify QTLs in iPSCs. From a statistical perspective, this may seem counterintuitive, but these results are consistent with previous work showing that, while inter-individual variation in gene expression was reduced in iPSCs compared with LCLs, a high proportion of the variation in iPSCs segregated by individual (Thomas et al. 2015). Taken together, these results suggest that a lower proportion of the regulatory variation in differentiated tissues is under genetic control—consistent with the notion that differentiated tissues can tolerate a high degree of gene expression variability (i.e., canalization)—while pluripotent cells are more tightly regulated. Interestingly, we find the increased variation in differentiated cell types is also associated with a slight but significant increase in correlated expression levels across genes (Supplemental Materials), further highlighting the level of regulatory control in iPSCs.

One of our goals was to use a multi-omics approach to better identify genetic variants with cell-type–specific regulatory effects in LCLs, iPSCs, and iPSC-CMs. To this end, we identified a list of iPSC- and LCL-specific eQTLs. We further identified chromatin features that are associated with cell-type–specific and shared eQTLs across all three cell types (Supplemental Fig. S19). As we collected multiple sources of data, we were also able to identify putative mechanisms that drive such eQTLs. In particular, the chromatin accessibility data allowed us to identify cell-type–specific caQTLs in LCLs, iPSCs, and iPSC-derived cardiomyocytes. We estimated that 80% of the cell-type–specific caQTLs affected loci with cell-type–specific accessibility patterns, whereas the remaining 20% are affected loci where chromatin was accessible in multiple cell types. We hypothesize that cell-type–specific caQTLs within loci accessible in multiple cell types are likely driven by cell-type–specific TF binding, although more work is needed to determine the transcription factors involved in such cases and whether these loci correspond to chromatin targeted by pioneer TFs.

A major goal of human genetics is to predict the impact of genetic variants on phenotype. Machine learning methods and, in particular, deep learning have become promising tools for identifying important features in genomics data sets (Libbrecht and Noble 2015). The chromatin accessibility data generated in this study seemed particularly amenable to such techniques. Thus, we developed a deep learning tool, OrbWeaver, in an attempt to identify sequence features predictive of open chromatin. OrbWeaver allowed us to identify TFs with known cell-type–specific effects. In the future, we expect that OrbWeaver, or similar approaches, will help us identify additional TFs underlying chromatin accessibility changes in response to functional perturbations. More interestingly, we found that OrbWeaver can accurately predict the direction of effect of cell-type–specific caQTLs. We acknowledge that, while the prediction accuracy is high for SNPs known to be caQTLs, predicting the effect of genetic variants on chromatin accessibility remains highly challenging.

Finally, we demonstrate the utility of iPSC-derived cells for the study of regulatory phenotypes. While iPSCs have been used to model a number of human diseases (Yagi et al. 2011; Choi et al. 2013; Liang et al. 2013; Miller et al. 2013; Aflaki et al. 2014; Pashos et al. 2017; Cayo et al. 2017), there is a limited amount of work demonstrating their ability to model regulatory phenotypes (Alasoo et al. 2017). iPSC-CMs recapitulate gene expression patterns observed in primary heart tissue obtained from the GTEx Consortium, and eQTLs identified in iPSC-CMs are also enriched among eQTLs identified in primary heart tissue (Supplemental Fig. S8). These observations suggest that iPSC-derived cells not only recapitulate the broad regulatory profile of their in vivo counterparts but also mirror tissue-specific functional genetic variation. These results have important implications as many disease-associated genetic variants are thought to have context- and cell-type–specific effects. For example, we found an iPSC-CM-specific enrichment of variants involved in cardiac diseases. A next goal is to identify mechanisms by which genetic variants affect disease by inducing iPSC-derived cells into different disease-relevant contexts.

Ultimately, we believe that our iPSC lines will be of great value. In particular, future studies using this panel of iPSCs will be able to assay dynamic gene regulation by characterizing gene expression during differentiation, in multiple cell types from the same individuals, and in terminally differentiated cell types subjected to experimental perturbations. The move toward dynamic studies of gene regulation in disease-relevant tissues will help to elucidate mechanisms underlying complex disease that were previously difficult or impossible to study. The research presented here is a first step toward this goal.

## Methods

### Sample collection

After at least three passages in feeder-free conditions, iPSCs were passaged into a 10-cm culture dish. At near full confluence, cells were enzymatically dissociated and counted. After dissociation, all additional steps are performed on ice or in a temperature-controlled centrifuge. One 10-cm dish yields between 3 million and 15 million cells. From each line, 400,000 cells were divided into two tubes to be used for ATAC-seq (Buenrostro et al. 2013). The tagmentation step of the ATAC-seq protocol was performed immediately on the two cell pellets containing 200,000 cells each. The library preparation of ATAC-seq samples was done in larger batches at a later time. The remaining material was split among three tubes for RNA and DNA extractions. We isolated RNA and DNA using the Zymo dual extraction kits (Zymo Research) with a DNase treatment during RNA extraction (Qiagen) on a single cell pellet from each line. Fifty-base pair single-end RNA sequencing libraries were generated from extracted RNA using the Illumina TruSeq kit as directed by the manufacturer. Sequencing of samples was performed on an Illumina HiSeq 2500. Extracted DNA was bisulphite-converted and hybridized to the Infinium MethylationEPIC array (Illumina) at the University of Chicago Functional Genomics facility. A similar procedure (Supplemental Materials) was used to collect iPSC-CM samples.

### iPSC and iPSC-CM generation and characterization

We reprogrammed LCLs into iPSCs using an episomal reprogramming approach described previously (Okita et al. 2011; Burrows et al. 2016). Briefly, we transfected 1 million LCLs with 1 µg of oriP/EBNA1 PCXLE-based episomal plasmids that contain the genes *POU5F1*, *SOX2*, *KLF4*, *MYCL*, *LIN28*, and an shRNA against *TP53* (Supplemental Materials; Okita et al. 2011; Burrows et al. 2016). All iPSC lines were characterized for pluripotency and stability using the following criteria: (1) the ability of lines to differentiate to all three germ layers using the embryoid body (EB) assay; (2) all lines were karyotyped to search for large genomic rearrangements; and (3) PluriTest (Muller et al. 2011) was applied to gene expression data to assay pluripotency bioinformatically (Supplemental Materials). Differentiation from iPSCs to cardiomyocytes was performed using slight modifications of existing protocols (Supplemental Materials for more details; Lian et al. 2013; Burridge et al. 2014). All samples reported here were of a high purity (a median of 82% of cells of each individual express cardiac Troponin T) (Supplemental Materials).

### Molecular data processing

RNA-seq from LCLs (Lappalainen et al. 2013) and iPSCs were mapped using the STAR RNA-seq aligner (Dobin et al. 2013) standard settings and processed using WASP to filter out reads that map with allelic bias (van de Geijn et al. 2015). RNA-seq reads from cardiomyocytes were mapped using Subread (Liao et al. 2013), allowing for two mismatches, and were also filtered using WASP for biases in allelic mapping (Supplemental Materials).

Paired-end ATAC-seq reads were mapped using Bowtie 2 (Langmead and Salzberg 2012), allowing for two mismatches per read. After mitochondrial reads were removed, we once again remapped all nuclear reads using the WASP to remove reads that map with allelic bias. We then removed all duplicate fragments (duplicates of both read pairs) and reads with a mapping quality (MAPQ) less than 10.

### Regulatory variation in iPSCs

To quantify the regulatory variation in gene expression, chromatin accessibility, and DNA methylation levels, we calculated the average square distance from the mean for each individual *n* as defined as:

$$V_{n}=\;\frac{N}{L(N-1)}\;\overset{L}{\underset{l=1}{\sum }}\frac{{\left(\chi_{nl}-\;\bar{\chi }\right)}^{2}}{{\bar{\chi }}^{2}}$$

for loci *l* and locus mean $\bar{\chi }$.

### QTL mapping

We used the following approaches to identify molecular QTLs in our study:

- eQTLs in iPSCs and LCLs: We transformed expression levels to a standard normal within each individual. We next accounted for unknown confounders by removing principal components from the LCL (15 PCs) and iPSC (10 PCs) data. Genotypes were obtained using impute2 as described previously (Li et al. 2016). We only considered variants within 50 kb of genes. To identify association between genotype and gene expression, we used FastQTL (Ongen et al. 2016). After the initial regression, a variable number of permutations were performed to obtain a gene-wise adjusted *P*-value (Ongen et al. 2016). To identify significant eQTLs, we used Storey's *q*-value (Storey and Tibshirani 2003) on the adjusted *P*-values. Genes with a *q*-value less than 0.1 are considered significant.
- eQTLs in iPSC-CMs: We used the combined haplotype test (CHT) (van de Geijn et al. 2015) to identify eQTLs using both regression and allelic imbalance tests in combination. We focused on variants within 25 kb of a gene. Following the procedure outlined by the authors (Storey and Tibshirani 2003), we performed the CHT and one permutation of the CHT. We noted that our tests were not well calibrated, owing to the small number of samples. We therefore identified significant SNPs by performing Storey's *q*-value correction (Storey and Tibshirani 2003) on the null data. We then identified the largest *P*-value in the null data with a *q*-value less than 0.1. We used this *P*-value as a threshold in the nonpermuted data to identify significant eQTLs.
- meQTLs in iPSCs and LCLs: We transformed methylation levels to a standard normal within each individual, and principal components were removed to account for unknown confounders (iPSC: six PCs removed; LCLs: five PCs removed). In accordance with previous work, genetic variants within 3 kb of a CpG were tested for associations with methylation levels. Methylation QTLs were identified using the FastQTL software (Ongen et al. 2016) following the procedure described above.
- caQTLs in all cell types: We pooled the ATAC-seq data for 12 individuals from whom we have ATAC-seq data in all three cell types to create a chromatin accessibility track for each cell type (Supplemental Materials for more details). We then used WASP to identify caQTLs in all cell types separately.
- distal caQTLs in LCL and iPSCs: We used ATAC-seq data from iPSCs (*n* = 58) and DNase-seq data from LCLs (*n* = 68). Chromatin accessibility levels were fit to a standard normal across individuals and qqnormed within individual (Degner et al. 2012). Principal components were removed to account for unknown confounders (iPSCs: one PC removed; LCLs: two PCs removed). Associations between genetic variants within 500 kb of a peak and chromatin accessibility levels were identified using FastQTL (Ongen et al. 2016).

### Peak calling using MACS2

To identify a stringent set of accessible regions in our cell types, we used MACS2 (Zhang et al. 2008; https://github.com/taoliu/MACS) to call peaks in all individual ATAC-seq samples separately:

macs2 callpeak --treatment bamfile --gsize hs --format BAMPE -q 0.01

We next merged all peaks for each individual sample by cell type, requiring that a peak has a 15× fold change enrichment over background signal.

### Estimating QTL sharing

Storey and Tibshirani (2003) developed a method to estimate the true proportion of null statistics from a given *P*-value distribution. This metric (π_0_) can be used to calculate the proportion of significant tests from a *P*-value distribution by taking 1 − π_0_ (π_1_). Here, we calculate π_1_ for eQTLs, caQTL, and meQTLs between cell types. To obtain a better estimate of the true sharing, we generated π_1_ statistics for a range of stringencies. Specifically, for eQTLs and caQTLs, we calculated π_1_ cumulatively from the top 150 most significant genes/loci to the top 2000 most significant genes/loci in intervals of 25 genes/loci. For meQTLs, we calculated π_1_ from the top 500 CpGs to the top 10,000 CpGs in intervals of 100 CpGs. This method allows us to see sharing across a wide space of stringencies.

### Linking cell-type–specific caQTL to eQTL signal

We used a one-sided Fisher's exact test to determine the level of significance at which the number of iPSC-specific caQTLs that are also iPSC eQTLs is greater than the number of LCL-specific caQTLs that are also iPSC eQTLs (and vice versa). This yielded a *P*-value of 4.7 × 10^−5^ and 0.01 for the two comparisons, respectively. This result is robust with respect to various thresholds at which we defined LCL and iPSC eQTLs (e.g., 10^−2^, 10^−3^, 10^−4^, 10^−5^). To obtain a set of iPSC-specific caQTLs that also affect expression of distal genes, we identified cell-type–specific caQTLs SNPs that were also associated with expression level of a nearby gene (100 kb) in iPSC with a nominal *P*-value of, at most, 0.001.

### GWAS signal enrichments in gene expression data

We used RolyPoly, a polygenic method that identifies trait-involved cell types by analyzing the enrichment of GWAS signal in cell-type–specific gene expression genome-wide (Calderon et al. 2017). To compute disease heritability enrichments in chromatin marks and our ATAC-seq peaks, we used stratified LDscore regression (Supplemental Materials; Finucane et al. 2015).

### Neural network models for chromatin accessibility

To predict the chromatin activity of a genomic locus across three cell types (iPSC, LCL, and iPSC-CM) from the DNA sequence, we used a one-hot encoding of the reference DNA sequence of length 500 bp centered at the locus as the input to the neural network model. The input layer therefore consists of 4 × 500 binary-valued variables. The output of a neural network model is a categorical variable O ∈{1,…, 7} where the values of the variables denote the following: 1 if open in iPSC-CM alone, 2 if open in LCL alone, 3 if open in iPSC-CM and LCL, 4 if open in iPSC alone, 5 if open in iPSC and iPSC-CM, 6 if open in iPSC and LCL, 7 if open in all three cell types.

We used the sigmoid activation function to model the probability of the categorical variable in the output layer. The architecture of our neural network, OrbWeaver, can be found in Supplemental Materials. The filters of the first convolutional layer in OrbWeaver were kept fixed to log-transformed position weight matrices of 1320 human transcription factors. For each TF, we used PWMs curated from two sources—TRANSFAC (Matys et al. 2006) and HT-SELEX (Supplemental Materials; Jolma et al. 2013).

To train our neural network, we used a training set of 282,088 loci to learn the parameters of each model using ADADELTA (Zeiler 2012).

We queried and interpreted the importance of each of the factors in predicting active chromatin belonging to one of the seven categories by fixing the filters in the first convolutional layer to known TF PWMs. We computed importance scores using DeepLIFT (Shrikumar et al. 2016), and for each of the seven categories, we used loci belonging to that category if the model correctly predicted their category. For each locus, we calculated DeepLIFT scores on the input with respect to each filter in the first convolutional layer; this gives us a score for each TF at each position in the locus (Supplemental Methods).

To predict the effects of genetic variation on chromatin accessibility at loci tested for caQTLs, we first used qtlBHM, a Bayesian hierarchical model (https://github.com/rajanil/qtlBHM), without any annotation to compute the probability that a locus is a caQTL (π_l_) and the probability that a SNP is the causal variant for a locus conditional on the locus being a caQTL (π_s_). Restricting to loci with π_l_ > 0.99 and π_s_ > 0.99, using a 500-bp window centered at the causal variant of each such locus, we computed the OrbWeaver prediction at each of the 240 haplotypes (corresponding to 120 YRI individuals). Partitioning the haplotypes based on the alleles of the causal SNP, we then computed the difference in the median prediction of chromatin activity between the reference and alternate alleles for each of the three cell types.

### Software availability

OrbWeaver, our deep learning software, is available freely at https://github.com/rajanil/OrbWeaver and as a Supplemental file.

## Data access

All data from this study have been submitted to the Gene Expression Omnibus (GEO; www.ncbi.nlm.nih.gov/geo/) under accession no. GSE89895 and at http://eqtl.uchicago.edu/yri_ipsc/. Other accession numbers can be found in Supplemental Table S7.

## Supplementary Material

Supplemental Material
