# Supplementary material for: Impact of regulatory variation across human iPSCs and differentiated cells
Source: Genome Res. 2018 Jan;28(1):122–31. doi: 10.1101/gr.224436.117 (PMC5749177; doi:10.1101/gr.224436.117)
Supplement: Supplemental Material [file supp_28_1_122__index.html]

Impact of regulatory variation across human iPSCs and differentiated cells — Supplemental Material 

# Impact of regulatory variation across human iPSCs and differentiated cells

## Supplemental Material

- Supplemental\_OrbWeaver-master.zip
- Supplemental\_Information.pdf
