# Supplementary material for: Impact of regulatory variation across human iPSCs and differentiated cells
Source: Genome Res. 2018 Jan;28(1):122–31. doi: 10.1101/gr.224436.117 (PMC5749177; doi:10.1101/gr.224436.117)
Supplement: Supplemental Material [file supp_gr.224436.117_Supplemental_Information.pdf]

Supplementary Information for

Impact of regulatory variation across human iPSCs and differentiated cells

Nicholas E. Banovich[1,‡*], Yang I. Li[2*], Anil Raj[2*], Michelle C. Ward[1,3], Peyton Greenside[4], Diego Calderon[4], Po Yuan Tung[1,3], Jonathan E. Burnett[1], Marsha Myrthil[1], Samantha M. Thomas[1], Courtney K. Burrows[1], Irene Gallego Romero[1,‡], Bryan J. Pavlovic[1], Anshul Kundaje[2], Jonathan K. Pritchard[2,5,6,†], Yoav Gilad[1,3,†]

[1]Department of Human Genetics, University of Chicago, Chicago, IL, USA.

[2]Department of Genetics, Stanford University, Stanford, CA, USA.

[3]Department of Medicine, University of Chicago, Chicago, IL, USA.

[4]Department of Biomedical Informatics, Stanford University, Stanford, CA, USA.

[5]Department of Biology, Stanford University, Stanford, CA, USA.

[6]Howard Hughes Medical Institute, Stanford, University, Stanford, CA, USA.

[‡]Present address. Translational Genomics Research Institute, Phoenix, AZ, USA (N.E.B.); Centre for Systems Genomics, University of Melbourne, Parkville, Australia (I.G.R.).

[*]These authors contributed equally to this work.

[†] **Corresponding author. Email: pritch@stanford.edu (J.K.P.); gilad@uchicago.edu (Y.G.).**

Contents

1 iPSC generation 5

2 iPSC characterization 5

3 iPSC-derived cardiomyocyte differentiation 6

4 LCL growth 7

5 Sample Collection 7

6 Molecular data processing 8

6.1 RNA-seq processing 8

6.2 ATAC-seq processing 9

6.3 DNase-seq processing 9

6.4 Methylation array processing 10

7 Reduced regulatory variation in iPSCs 10

8 QTL mapping 12

8.1 Identifying eQTLs 12

8.2 Identifying meQTLs 12

8.3 Identifying caQTLs 13

8.3.1 Peak calling using MACS2 (Fig. 2B) 14

9 Estimating QTL sharing 15

10 Identifying cell type specific and shared eQTLs/caQTLs 15

10.1 Linking cell-type specific caQTL to eQTL signal (Fig. 2A) 15

10.2 Accessibility at cell-type specific caQTLs (Fig. 2B) 16

10.3 Accessibility at LCL and iPSC caQTLs (Fig. 2E) 17

11 Hierarchical model to fine-map causal eQTLs and caQTLs 17

12 GTEx data 18

13 GWAS signal enrichments in gene expression data (Fig. 4A) 19

14 Stratified LDscore regression (Fig. 4B) 19

15 Neural network models for chromatin accessibility 20

15.1 Model components 20

15.2 Model architectures 21

15.3 Model learning 22

15.4 Identifying key transcription factors for each category of chromatin activity 23
15.5 Predicting effects of genetic variation on chromatin accessibility 23

16 Raw and processed data availability **23**

List of Tables

S1 Purity of iPSC-CMs. 7
S2 Final sequencing read depths (nuclear) for chromatin accessibility 11
S3 Counts of QTLs for each molecular phenotype in each cell type 13
S4 Contingency table for cell type specific caQTLs that are LCL eQTLs at p-value $< 10^{-3}$. . . . 17
S5 Contingency table for cell-type-specific caQTLs that are iPSC eQTLs at p-value $< 10^{-3}$. . . . 17
S6 iPSC-specific caQTLs that also affect expression of distal genes 17
S7 Transcription factors predictive of chromatin activity 24

List of Figures

S1 Induced pluripotent stem cell quality control 31
S2 Quantifying library quality using proportion of reads in promoters 32
S3 Quantifying library quality using chromatin accessibility around TSS 33
S4 Comparing the fragment-size distributions between ATAC-seq libraries. 34
S5 Quantifying ATAC-seq read loss at various data processing steps. 35
S6 Molecular data QC 36
S7 Variation of iPSC, LCLs, and iPSC-CM molecular traits. 37
S8 Enrichment of cardiomyocyte eQTLs among GTEx eQTLs. 38
S9 Quantifying sharing of QTLs. 38
S10 Concordance of caQTL and eQTL effect size direction 39
S11 Concordance of caQTL and eQTL effects 40
S12 Comparison of absolute effect sizes of eQTLs and caQTL-eQTLs 41
S13 Examples of distal caQTLs affecting various genomic interactions. 42
S14 Examples of shared caQTLs in LCLs and IPSCs. 43
S15 Chromatin accessibility signal at LCL-specific caQTLs. 44
S16 Deep neural network architectures for predicting chromatin activity 45

S17 Comparing the accuracy of neural network architectures. 46
S18 Predicting genetic effects on chromatin activity using neural network models. 47
S19 Quantifying eQTLs explained by chromatin annotations. 48

Supplementary Methods

1 iPSC generation

We reprogrammed LCLs into iPSCs using an episomal reprogramming approach described previously (*Okita et al., 2011, Burrows et al., 2016*). Briefly, we transfected 1 million LCLs (Amaxa^{TM} Nucleofector^{TM} Technology; Lonza) with 1ug of oriP/EBNA1 PCXLE based episomal plasmids that contain the genes OCT3/4, SOX2, KLF4, L-MYC, LIN28, and an shRNA against p53 (*Okita et al., 2011, Burrows et al., 2016*). Cells were cultured in suspension for seven days after transfection in hESC media (DMEM/F12 supplemented with 20% KOSR, 0.1mM NEAA, 2mM GlutaMAX, 1% Pen/Strep, 0.1% 2-Mercaptoethanol, 25ng/ul of bFGF and .5mM NaB). On the 8th day we plated a range of 8,000 - 32,000 transfected cells per well in a 6-well plate coated with gelatin and seeded with irradiated CF1 mouse embryonic fibroblasts (MEF). Four days after the initial plating NaB was removed from the hESC media. Within 21 days colonies were visible and manually passaged onto a freshly prepared gelatin plate seeded with MEF. Manual passaging continued weekly for ten weeks. After ten passages of growth cells were expanded and at least ten stocks of cells were cryopreserved. Colonies were then transitioned to feeder-free conditions and cultured for at least an additional three passages before collecting cell pellets for analysis. Feeder-free cultures were grown using 0.01mg/cm2 (1:100) hESC-grade Matrigel and Essential 8 (E8) media. Feeder-free passaging is enzymatic rather than manual and was performed using DPBS supplemented with 0.5mM EDTA.

2 iPSC characterization

All iPSC lines were characterized for pluripotency and stability using three methods. First, we confirmed the ability of lines to differentiate to all three germ layers using the embroyid body (EB) assay. Lines were manually dissociated from their culture dish in large pieces. This material was then cultured in a suspension plate using the hESC media described above without bFGF for one week, while dense spherical EBs form. EBs are then plated into 12 well plates with gelatin and cultured in EB medium (DMEM supplemented with 10% FBS, 0.1mM NEAA, 2mM GlutaMAX) for one week. EBs in each well were then immunostained for cell types from all three germ layers Supplementary Figure S1. Next, all lines were karyotyped to search for large genomic rearrangements Supplementary Figure S1. Lines were karyotypes by the WiCell Research Institute (Madison, WI). Only one line, 19128, showed large genomic rearrangements that were not known rearrangements segregating in the population. The rearrangement observed in this line is a hallmark rearrangement of follicular lymphoma and thus was likely present in LCLs rather than

a result of the reprogramming process. Finally, a classifier, PluriTest (*Muller et al., 2011*) was applied to gene expression data (Illumina HumanHT-12 array) to assay pluripotency bioinformatically. The classifier compares gene expression levels from uncharacterized lines to a "gold standard" panel of embryonic stem cells and iPSCs. Two metrics are obtained from this method, a pluripotency score and a novelty score. The pluripotency score represents goodness of fit of canonical pluripotency genes in the sample. The novelty score represents the deviance of non-pluripotency genes in the sample. All of the lines here pass the suggested empirical threshold (Supplementary Figure S1).

3 iPSC-derived cardiomyocyte differentiation

Differentiation from iPSCs to cardiomyocytes was performed using slight modifications of existing protocols (*Lian et al., 2013, Burridge et al., 2014*). Specifically, iPSCs cultured under feeder-free conditions were seeded onto a 10 cm dish three to five days prior to differen- tiation. When cells were 70-100% confluent E8 media was replaced with 'heart media' (RPMI (14-040-CM, Thermo Fisher Scientific) supplemented with B-27 Supplement, minus insulin (A1895601, Thermo Fisher Scientific), 2mM GlutaMAX (35050-061, Thermo Fisher Scientific), and 100mg/mL Pennicllin/Streptomycin (30002Cl, Corning)) with the addition of 1:100 Matrigel (35427, Corning) and 12uM of the GSK-3 inhibitor CHIR99021 trihydrochloride (4953, Tocris,) which activates WNT signaling (day 0) (*Lian et al., 2013*). 'Heart media' was replaced 24 hours later (day 1). After an additional 48 hours media was replaced with new 'heart media' containing 2uM of the WNT inhibitor Wnt-C59 (5148, Tocris) (*Lian et al., 2013*) (day 3). Cells were cultured in the media with Wnt-C59 for 48 hours. Regular 'heart media' changes were performed from day 5 to day 14. Clusters of spontaneously beating cells were typically visible between 7 and 12 days. On day 14 heart media was replaced with 'lactate media' (RPMI without glucose (11879, Gibco), 75 mg/ml human recombinant albumin (A0237, Sigma), 213 ug/ml L-ascorbic acid 2-phosphate (sc228390, Santa Cruz Biotechnology), 5mM Sodium L-lactate (L7022, Sigma), and 100mg/mL Pennicillin/Streptomycin) 24930130. The 'lactate media' purifies cardiomyocytes using metabolic selection whereby the majority of non-cardiomyocyte cells cannot use lactate as their primary source of energy, leaving a culture significantly enriched for cardiomyocytes (*Tohyama et al., 2013*). Every other day 'lactate media' changes were performed until day 20. By day 20 the cells had generally formed into large three-dimensional sheets of beating cells. To make a more uniform sheet of cells we dissociated the cultures using 0.05% trypsin (25-053-Cl, Thermo Fisher Scientific) and replated cells into six-well plates at a density of 1.5 million cells per well. Cells were then cultured in 'galactose media' (DMEM without glucose (A14430-01, Thermo Fisher Scientific), 1.7 mg/mL galactose (G5388, Sigma), 1mM Na pyruvate

(11360-070, Gibco), 5mM HEPES (SH3023701, Thermo Fisher Scientific), 2mM GlutaMax, 10%FBS (4360-500, J R Scientific), and 100mg/mL Pennicillin/Streptomycin) to help to mature cardiomyocytes by forcing them to undergo aerobic metabolism (*Wang et al., 2014, Marroquin et al., 2007*). Regular media changes with 'galactose media' continued for the duration of the experiment. After an additional five days (day 25) cells were moved to an incubator at physiological oxygen levels (10%). Two days later (day 27) cells were subjected to electrical stimulation at 6.6 V/cm, 2 ms and 1 Hz (C-PaceEP Cell Culture EP Stimulator, IonOptix) for four days to help further mature the cells (*Chan et al., 2013*) and standardize beating rate across wells and lines. Cardiomyocytes were harvested on day 31/32. To establish the purity of iPSC-CMs flow cytometry was performed using a cardiac-specific marker, cardiac Troponin T (564767, BD Biosciences). All samples reported here were of a high purity (a median of 82% of cells of each individual express cardiac Troponin T; see table S1)

Line	Purity
18499	80.6
18505	89.7
18511	76
18520	93.5
18852	75.4
18855	85
18870	87.1
18912	90
19098	96.9
19101	47.9
19108	81.4
19116	82.9
19128	40
19160	79.8

Table S1: Purity of iPSC-CMs as measured by flow cytometry using the cardiac troponin T marker.

4 LCL growth

LCLs were grown in cells in standard media (RPMI1640, 15%FBS, 0.1mM NEAA, 2mM GlutaMAX) and maintained at a density of between 200,000-500,000 viable cells/ml.

5 Sample Collection

After at least three passages in feeder-free conditions iPSCs were passaged into a 10cm culture dish. At near full confluence cells were enzymatically dissociated and counted. After dissociation all additional steps are performed on ice or in a temperature controlled centrifuge. One 10cm dish yields between 3 million

and 15 million cells. From each line 400,000 cells were divided into two tubes to be used for ATAC-seq (*Buenrostro et al., 2013*). The tagmentation step of the ATAC-seq protocol was performed immediately on the two cell pellets containing 200,000 cells each. The library preparation of ATAC-seq samples was done in larger batches at a later time. The remaining material was split between three tubes for RNA and DNA extractions. We isolated RNA and DNA using the Zymo dual extraction kits (Zymo Research) with a DNase treatment during RNA extraction (Qiagen) on a single cell pellet from each line. 50 bp single-end RNA sequencing libraries were generated from extracted RNA using the Illumina TruSeq kit as directed by the manufacturer. Sequencing of samples was performed on an Illumina 2500. Extracted DNA was bisulphite-converted and hybridized to the Infinium MethylationEPIC array (Illumina) at the University of Chicago Functional Genomics facility.

iPSC-CMs were collected on ice using manual dissociation. We isolated RNA and DNA using the Zymo dual extraction kits (Zymo Research) with a DNase treatment during RNA extraction (Qiagen) on a single cell pellet from each line. RNA sequencing libraries were generated from extracted RNA using the Illumina TruSeq kit as directed by the manufacturer. 50bp single-end RNA-seq was performed on an Illumina 2500. A pellet containing 200,000 cells was used immediately for the tagmentation step of the ATAC-seq protocol. The library preparation of ATAC-seq samples was done in larger batches at a later time. LCLs were collected by pelleting 200,000 cells in a temperature controlled centrifuge. The tagmentation step of the ATAC-seq protocol was performed immediately on a pellet containing 200,000 cells. The library preparation of ATAC-seq samples was done in larger batches at a later time.

6 Molecular data processing

6.1 RNA-seq processing

RNA-seq from LCLs (*Lappalainen et al., 2013*) and iPSCs were mapped using the STAR RNA-seq aligner (*Dobin et al., 2013*) standard settings and processed using WASP to filter out reads that map with allelic bias (*van de Geijn et al., 2015*). RNA-seq reads from cardiomyocytes were mapped using Subread allowing for two mismatches and were also filtered using WASP for biases in allelic mapping. Reads overlapping SNPs were remapped to reduce reference bias as described previously (*van de Geijn et al., 2015*). Only reads with a MAPQ greater than 10 were retained. Read depths are provided in Table S2.

To determine the organs and cell-types with regulatory profiles most similar to our LCLs, iPSC, and iPSC-CMs **(Fig. 1B)**, we used kallisto (*Bray et al., 2016*) to quantify transcript (gencode v19) isoform abundances

in our samples (LCLs, iPSC, iPSC-CM) along with samples from GTEx (Heart – Atrial Appendage, Heart – Left Ventricle, Bone Marrow, Pancreas, Testis, Brain, Kidney, Colon, Lung, Skin, and Blood) (*Ardlie et al., 2015*) and ENCODE (H1-ESC, LCLs) (*Dunham et al., 2012*). We next identified a set of organ-regulated genes by using five samples for each organ. To do this, for each gene, we compared the distribution of transcript abundance in the organ to abundance of the transcript in all other organs using a Mann-Whitney U test. We then took 500 genes with the most differentially expressed transcripts (according to the Mann-Whitney U p-values) for each organ, which resulted in 5,000 organ-regulated genes. Lastly, we computed the Spearman correlation of the transcript abundances for these 5,000 genes for each sample pair.

6.2 ATAC-seq processing

Paired end ATAC-seq reads were mapped using bowtie2 allowing for two mismatches per read. The ATAC-seq protocol works by randomly inserting sequencing adapters into open chromatin via a tagmentation enzyme. One unfortunate side effect of this procedure is a high enrichment of reads originating from mitochondrial DNA (between 25%-75% of reads). Yet, we chose not to pursue an enrichment approach prior to sequencing, in order to avoid introducing additional variation to the nuclear gene expression data. After mitochondrial reads were removed, we re-mapped all nuclear reads using the WASP pipeline to filter out reads that did not uniquely map to the same genomic location after accounting for genetic variation (*van de Geijn et al., 2015*). We then removed all duplicate fragments (duplicates of both read pairs) and reads with a mapping quality (MAPQ) less than 10. Each mate represents an independent tagmentation event and therefore, after mapping and duplicate removal, reads were treated as single end in all future analyses. The quality of our ATAC-seq libraries in all three cell lines were very similar to that of ATAC-seq data in the LCL GM12878 cell line (*Buenrostro et al., 2013*), ATAC-seq data in the H1hesc cell line (courtesy of the Greenleaf Lab) and DNase-seq data in the YRI LCL cell lines (*Degner et al., 2012*) (see figs. S2, S3, and S4). Final read depths are provided in Table S2 and the fractions of reads filtered out after each processing step are illustrated in Supplementary Figure S5.

6.3 DNase-seq processing

Previously collected DNase-seq from LCLs was used to assay chromatin accessibility. Reads were mapped using a custom mapper, which has been previously described in depth (*Pique-Regi et al., 2011*). In this study counts per base were obtained directly from a previous study (*Degner et al., 2012*).

6.4 Methylation array processing

Methylation levels were assayed using the Infinium MethylationEPIC array (Illumina) in iPSCs and the Infinium HumanMethylation450 array (Illumina) in LCLs. Methylation data from LCLs were obtained from a previous study (*Banovich et al., 2014*). In iPSCs a number of steps were taken to ensure high quality data. First, to enable accurate quantification of methylation levels all probes that contained a SNP with a MAF greater than 5% in the population were removed. Next, we removed all CpGs that were not detected in at least 75% of individuals. CpGs on the X or Y chromosome were removed.

7 Reduced regulatory variation in iPSCs

To quantify the regulatory variation in gene expression, chromatin accessibility, and DNA methylation levels, we calculated the average square distance from the mean for each individual n as defined as:

$$V_n = \frac{N}{L(N-1)} \sum_{l=1}^{L} \frac{(x_{nl} - \bar{x})^2}{\bar{x}^2}$$

for loci l and locus mean $\bar{x}$. iPSCs have consistently lower variation compared with LCLs and iPSC-CMs across all three regulatory phenotypes (Supplementary Figure S7), and significantly lower variation in gene expression (LCLs: $p < 10^{-6}$; iPSC-CMs: $p < 10^{-5}$; Mann–Whitney U) and DNA methylation (LCLs; $p < 10^{-11}$) levels. Chromatin accessibility was significantly lower in iPSCs compared to iPSC-CMs ($p = 2 \times 10^{-4}$) but not LCLs ($p = 0.42$).

Differentiations from iPSCs to iPSC-CMs remains an imperfect process. Thus, the observed increase in expression variability could be due to technical rather than biological variation. However, we found no significant correlation between the average square distance from the mean and the purity of the iPSC-CM culture. Furthermore, when considering genes specific to cardiomyocytes we observe an increase in the average square distance from the mean (0.53 vs 0.26). Finally, our results are replicated in primary heart tissue from GTEx, where the mean average square distance from the mean across individuals is slightly but insignificantly higher in primary heart (0.36) compared to iPSC-CMs (0.26; $p = 0.1251$).

We compared the gene-wise correlation levels of the top 10,000 genes within each cell-type. While all three samples are normally distributed around zero, we observe a slight increase in mean gene-wise correlation in iPSC-CMs (0.08) and LCLs (0.02) compared to iPSCs (0.01).

Sample	iPSC (ATAC)	LCL (ATAC)	LCL (DNase)	iPSC-CM (ATAC)
NA18486	32659004	-	42320431	-
NA18489	18879326	-	-	-
NA18498	30441944	-	42626294	-
NA18499	21191168	-	42508766	6198578
NA18501	35810874	15674600	33492742	-
NA18502	18752114	14083042	27546193	-
NA18504	-	36825244	27849233	-
NA18505	31937588	5371198	27446048	27664492
NA18507	-	-	74207029	-
NA18508	37029474	-	45294967	-
NA18510	15300428	-	46266665	-
NA18511	10675520	10560598	34325288	14151550
NA18516	-	-	28573172	-
NA18517	25455280	-	30218325	-
NA18519	17595712	32703926	44023326	-
NA18520	18571190	7302724	23360405	13295658
NA18522	56216208	7823794	23126812	-
NA18852	30521566	21607142	45901061	17157142
NA18853	11558304	-	29581654	-
NA18855	9065570	19680684	33802245	11861486
NA18856	13259732	-	30704705	-
NA18858	30453734	-	30070868	-
NA18859	13862852	-	29473448	-
NA18861	2635618	-	80235013	-
NA18862	19335654	-	32613319	-
NA18870	12410264	3287592	29196263	26946240
NA18907	26458794	-	44650582	-
NA18909	-	-	47794281	-
NA18912	23656490	87500262	27791308	12107644
NA18913	35096114	-	35757428	-
NA18916	-	-	31784568	-
NA19092	28648676	-	35404700	-
NA19093	3270958	-	13815173	-
NA19098	40165190	29180170	38472816	5325082
NA19099	40707618	-	29772954	-
NA19101	-	13478794	35503543	111719728
NA19102	45989426	6993378	-	-
NA19108	34009578	15842716	-	90819360
NA19114	4488602	-	48895531	-
NA19116	34003684	29583136	27680918	9386758
NA19119	15494768	-	29855656	-
NA19127	28119164	-	36647791	-
NA19128	28142480	34435242	-	26961610
NA19130	39817074	-	28993557	-
NA19131	-	-	35976533	-
NA19138	33720264	-	38512510	-
NA19140	15555202	-	34856598	-
NA19141	-	-	26834843	-
NA19143	22009676	-	35355157	-
NA19144	8856202	-	38393641	-
NA19147	-	-	48947479	-
NA19152	32112704	-	35249757	-
NA19153	14407548	-	30221567	-
NA19159	46076592	-	29652941	-
NA19160	26644496	4791876	36152805	46752622
NA19171	-	-	38884245	-
NA19190	25803656	-	36741524	-
NA19192	33147506	-	70691903	-
NA19193	10564818	-	39671681	-
NA19200	-	-	39434479	-
NA19201	-	-	36013821	-
NA19203	-	-	35304566	-
NA19204	29246864	-	40850302	-
NA19206	29077392	-	35333265	-
NA19207	21663330	-	35517782	-
NA19209	19455060	-	29632699	-
NA19210	10811076	-	-	-
NA19222	-	-	30295677	-
NA19223	-	-	34894059	-
NA19225	25977510	-	49138423	-
NA19239	10892712	-	108561800	-
NA19257	16167520	14729492	47206531	-

Table S2: Final sequencing read depths (nuclear) for chromatin accessibility

8 QTL mapping

8.1 Identifying eQTLs

To identify eQTLs in iPSCs and LCLs we transformed expression levels to a standard normal within each individual (iPSC: n= 59, LCL: n= 59). We next accounted for unknown confounders by removing principal components from the LCL (15 PCs) and iPSC (10 PCs) data. Genotypes were obtained using impute2 as described previously (*Li et al., 2016*). As in previous work, we were limited to examining putatively cis-acting genetic variants. Therefore, we only considered variants within 50kb of genes. To identify association between genotype and gene expression we used fastqtl (*Ongen et al., 2016*). After the initial regression, a variable number of permutations were performed to obtain a gene-wise adjusted p-value (*Ongen et al., 2016*). To identify significant eQTLs we used Storey's q-value (*Storey and Tibshirani, 2003*) on the adjusted p-values. Genes with a q-value less than 0.1 are considered significant.

The sample size of iPSC-CMs in this study was too limited to identify eQTLs using a standard regression model. We therefore used the combined haplotype test (CHT) (*van de Geijn et al., 2015*) to identify eQTLs. This method allowed us to identify eQTLs with small sample sizes by using both regression and allelic imbalance tests in combination. In this analysis, we focused on variants within 25kb of a gene. Following the procedure outlined by the authors (*Storey and Tibshirani, 2003*), we performed the CHT and one permutation of the CHT. We noted that our tests were not well calibrated, owing to the small number of samples. We therefore identified significant SNPs by performing Storey's q-value correction (*Storey and Tibshirani, 2003*) on the null data. We then identified the largest p-value in the null data with a q-value less than 0.1. We used this p-value as a threshold in the non-permuted data to identify significant eQTLs.

8.2 Identifying meQTLs

To identify meQTLs in iPSCs and LCLs, we transformed methylation levels to a standard normal within each individual (iPSC: n= 58; LCL: n= 64) and principal components were removed to account for unknown confounders (iPSC: 6 PCs removed; LCLs: 5 PCs removed). In accordance with previous work, genetic variants within 3kb of a CpG were tested for associations with methylation levels. Methylation QTLs were identified using the fastqtl software (*Ongen et al., 2016*) following the procedure described above. We inherently identified a larger number of meQTLs in iPSCs compared to LCLs due to the larger number of CpGs tested. However, we also compared only the CpGs shared across both arrays and found more meQTLs in iPSCs (n= 7,958; n= 5,738).

8.3 Identifying caQTLs

We first identified a comprehensive set of genomic loci that were open in iPSC, LCLs, or iPSC-CMs using the following approach. Focusing on the 12 individuals from whom we have data in all three cell types, we pooled the ATAC-seq data for all 12 individuals to create a chromatin accessibility track for each cell type; the data for each track corresponds to the count of transpositions (T_l) observed at each base pair. To account for differences in total read depth and differences in library quality (Supplementary Figure S2), we first converted the raw data track to a percentile track for each cell type as follows. Each position in the genome was assigned the total transposition count within the 100 bp window centered at that position, $S_l = \sum_{i=l-50}^{l+50} T_i$. Using an empirical distribution of S_l, each genomic position was assigned a percentile computed as $P_l = \frac{\sum_i \mathbb{I}[S_l > S_i]}{L}$, where $\mathbb{I}[\cdot]$ is the indicator function and L is the size of the genome. Given the percentile tracks for each cell type, we identified all genomic positions with $P_l \geq 90$ in at least one cell type, added a 50 bp flank to either end of each such position to create open chromatin intervals, and merged overlapping intervals to obtain $2,533,844$ loci.

Starting with these 2.5 million genomic loci, we focused our analysis on common SNPs (MAF $>$ 0.05) that were located within each locus to identify variants that are associated with and, likely, directly disrupt chromatin accessibility. For each cell type, we tested for association between these SNPs and the ATAC-seq signal across all individuals using the combined haplotype test in WASP (*van de Geijn et al., 2015*). Filtering out loci that did not contain a common SNP or did not have sufficient ATAC-seq reads overlapping the segregating SNPs, we ultimately tested 599,178 loci in iPSCs, 187,504 loci in LCLs, and 203,619 loci in iPSC-CMs. Using Storey's q-value (*Storey and Tibshirani, 2003*), we identified significant caQTLs at 10% FDR (see table S3).

To identify distal caQTLs, we used ATAC-seq data from iPSCs (n=58) and DNase-seq data from LCLs (n=68). Chromatin accessibility levels were fit to a standard normal across individuals and qqnormed within individual (*Degner et al., 2012*). Principal components were removed to account for unknown confounders (iPSCs: 1 PC removed; LCLs: 2 PCs removed). Associations between genetic variants within 500kb of a peak and chromatin accessibility levels were identified using fastqtl (*Ongen et al., 2016*).

cell-type	eQTL	caQTL	distal caQTL	meQTL
LCL	2556 (n=85)	3902 (n=20)	2260 (n=68)	5738 (n=64)
iPSC	1441 (n=57)	20370 (n=57)	2130 (n=57)	29782 (n=57)
iPSC-CM	517 (n=13)	4045 (n=14)	-	-

Table S3: Counts of QTLs for each molecular phenotype in each cell type. Sample sizes are listed in parentheses.

8.3.1 Peak calling using MACS2 (Fig. 2B)

To identify a more stringent set of accessible regions in our cell types, we used MACS2 to call peaks in all individual ATAC-seq samples separately:

```
macs2 callpeak --treatment bamfile --gsize hs --format BAMPE -q 0.01
```

We next merged all peaks for each individual samples by cell-type, requiring that a peak has a 15X fold change enrichment over background signal.

9 Estimating QTL sharing

Storey and Tibshirani developed a method to estimate the true proportion of null statistics from a given p-value distribution (*Storey and Tibshirani, 2003*). This metric (π_0) can be used to calculate the proportion of significant tests from a p-value distribution by taking $1-\pi_0$ (π_1). Here we calculate π_1 for eQTLs, caQTL, and meQTLs between cell types. To obtain a better estimate of the true sharing we generated π_1 statistics for a range of stringencies. Specifically, for eQTLs and caQTLs we calculated π_1 cumulatively from the top 150 most significant genes/loci to the top 2000 most significant genes/loci in intervals of 25 genes/lcoi. For meQTLs we calculated π_1 from the top 500 CpGs to the top 10,000 CpGs in intervals of 100 CpGs. As is clear from the density plots (Supplementary Figure S9), small deviations in threshold choice can create local valleys and peaks in sharing estimates. This method allows us to see sharing across a wide space of stringencies.

10 Identifying cell type specific and shared eQTLs/caQTLs

To identify cell type specific associations, we first removed loci that were tested in only one cell-type. Next, any locus with a significant association (even with a different lead variant) in both cell types was removed. An eQTL was considered cell-type specific if significant at an FDR of 10% in one cell type and a nominal p-value of greater than 0.05 in the second cell-type (as determined by fastqtl (*Ongen et al., 2016*)). QTLs were considered shared if they were significant with a p-value of less than 10^{-5} in both cell types.

To define cell type specific caQTLs, we required a WASP p-value smaller or equal to 5×10^{-4} in one cell-type, and a p-value greater than 0.05 in the second cell-type. **(Fig. 2A,B,E)**

10.1 Linking cell-type specific caQTL to eQTL signal (Fig. 2A)

We used a one-sided Fisher's exact test to determine the level of significance at which the number of iPSC-specific caQTL that are also iPSC eQTLs is greater than the number of LCL-specific caQTLs are also iPSC eQTLs (and vice-versa). This yielded a p-value of 4.7×10^{-5} and 0.01 for the two comparisons, respectively. This result is robust with respect to various thresholds at which we defined LCL and iPSC eQTLs (e.g. 10^{-2}, 10^{-3},10^{-4},10^{-5}).

We evaluated whether the effect of SNPs on chromatin accessibility and expression level of a nearby gene were generally correlated, and in the same direction, i.e. the allele that is associated with chromatin that is more open is also associated with increased in gene expression. To this end, we focused on the set of SNPs

that were associated to chromatin accessibility in LCLs and in IPSCs separately. Although we do no expect this analysis to yield different conclusions for this analysis, we note that the set of LCL caQTLs is much smaller than that of iPSC caQTLs (3,902 LCL caQTLS vs 20,370 iPSC caQTLs).

We first found that the level of concordance of the direction of effects depends on the choice of p-value association cutoffs for what we consider an eQTL or a caQTL (Figure S10). This is expected because the false discovery (and sign) rate changes with our p-value cutoff, with lower proportion of false discovery (and sign) at more stringent cutoffs. At the most stringent cutoffs, we estimated that the concordance levels were over 80% (Figure S10), suggesting that for a large majority of SNPs, the effects on chromatin accessibility is positively correlated with its effect on the expression levels of nearby genes.

Next, we quantified the levels at which magnitude of effects were correlated between caQTLs and eQTLs. Using a cutoff of $p < 10^{-3}$ as both caQTL and eQTL cutoff, for both cell types, we found that caQTL effect sizes could somewhat predict eQTL effect sizes or at least its direction of effects, as expected (linear regression $r^2 > 0.1$, $p < 10^{-9}$) (Figure S11). However, when we asked whether the absolute caQTL effect size could predict absolute eQTL effect, we found very weak or no significant predictive power ($p = 0.54$ for LCL QTLs, and $p = 0.02$ for iPSC QTLs). This suggests that the magnitudes of effect are not correlated between caQTLs and eQTLs.

Lastly, we asked whether eQTLs with an associated caQTL have a biased effect size distribution compared to eQTLs without associated caQTLs (Figure S12). To do this, we took all top eQTLs with $p < 10^{-3}$ and asked whether there was a difference between effect sizes of eQTLs with associated caQTLs ($\min p < 10^{-3}$) and the effect sizes of eQTLs without an associated caQTLs ($\min p \geq 10^{-3}$). Interestingly, we found that eQTLs that have associated caQTLs have a small, but significantly (Mann-Whitney U test $p < 10^{-4}$ for both LCLs and iPSCs) larger mean effect sizes than eQTLs that did not (LCL: 1.14-fold larger, iPSC: 1.13-fold larger). Although this may suggest that eQTLs that function through mechanisms associated with chromatin accessibility have generally stronger effects on gene expression levels (in contrast to post-transcriptional mechanisms), we can not rule the likely possibility that the eQTL false positive rates differ between the two sets, therefore biasing the effect size distributions.

10.2 Accessibility at cell-type specific caQTLs (Fig. 2B)

To determine accessibility at cell-type specific caQTLs, we centered all windows (1500bp total) and computed the mean number of ATAC-seq reads falling at each position (s), normalized by total read counts for each individuals. We then computed the $\log_2$ signal plus 1 ($\log_2(s+1)$) signal at each base, ranked all windows

by average signal across the window, and plotted in decreasing order of signal.

10.3 Accessibility at LCL and iPSC caQTLs (Fig. 2E)

We computed the mean accessibility at each open chromatin peak by summing over the number of reads falling into each peak and normalizing by total number of reads and peak length.

Cell-type	LCL-eQTL	not LCL-eQTL
LCL-specific caQTL	35	449
iPSC-specific caQTL	38	1309

Table S4: Contingency table for cell type specific caQTLs that are LCL eQTLs at p-value $< 10^{-3}$.

Cell-type	iPSC-eQTL	not iPSC-eQTL
LCL-specific caQTL	6	449
iPSC-specific caQTL	44	1309

Table S5: Contingency table for cell-type-specific caQTLs that are iPSC eQTLs at p-value $< 10^{-3}$.

To obtain a set of iPSC-specific caQTLs that also affect expression of distal genes, we identified cell-type-specific caQTLs SNPs that were also associated with expression level of a nearby gene (100kb) in iPSC with a nominal p-value of at most 0.001. We noted that a few iPSC-specific caQTL SNPs were also associated with gene expression level in LCLs despite showing no association for local chromatin accessibility. This observation is consistent with the possibility that some caQTLs SNPs within chromatin accessibility peaks are not the causal SNP. We note however that for the vast majority of SNPs, the observations are consistent with a distal effect of caQTLs on expression levels of nearby genes in the corresponding cell type (Fig. 2D).

List of iPSC-specific caQTLs with effects at distal genes
`http://eqtl.uchicago.edu/yri_ipsc/Table_caQTL_eQTL_iPSC.txt`

Table S6: iPSC-specific caQTLs that also affect expression of distal genes

11 Hierarchical model to fine-map causal eQTLs and caQTLs

Starting with summary statistics computed when identifying eQTLs and caQTLs, we used a Bayesian hierarchical model (`https://github.com/rajanil/qtlBHM`) to classify causal SNPs by incorporating annotations such as chromatin states and TF binding sites. The summary statistics used by the method are the effect size and standard error; the method is explained in detail elsewhere (*Li et al., 2016*).

For caQTLs, we computed the effect size (γ) from the allele-specific accessibility parameters reported by WASP (α and β) as $\gamma = \log \alpha - \log \beta$. To compute the standard error, we first computed the z-score from the reported p-value using a t-distribution with degrees of freedom as a function of the sample size. Since causal SNPs in caQTLs typically disrupt transcription factor binding sites, we first inferred binding for expressed TFs in the three cell types using msCentipede (`https://github.com/rajanil/msCentipede`) and used these inferred binding sites as genomic annotations in the hierarchical model. Inference under the model gives us the posterior probability that a locus is a caQTL, and the posterior probability that each SNP within the locus is the causal SNP conditional on the locus being a caQTL. Given these probabilities, we then computed the proportion of caQTLs that are explained by different TF binding annotations in each cell type.

For eQTLs, the effect sizes and standard errors were directly obtained from the output of fastqtl (*Ongen et al., 2016*) for iPSCs and LCLs, respectively, and computed from the output of WASP for iPSC-CMs. The output of qtlBHM included the inferred probabilities that a gene is an eQTL and the probability that a tested SNP is the causal SNP for a given eQTL gene. Among genes with posterior greater than 0.9 in one cell type, a gene was labeled as a shared eQTL if it had posterior greater than 0.9 in any other cell type, and it was labeled as a specific eQTL if it had posterior less than 0.1 in all other cell types. We were interested in characterizing the properties of shared and cell type-specific eQTLs, and to this end, we partitioned each genomic annotation into shared and cell type-specific annotations. For example, a particular genomic position was considered to be a shared promoter for iPSCs if the chromHMM state for that position is 'Promoter' in iPSCs and in at least one of the other two cell types, else the position is labeled an iPSC-specific promoter. For genomic annotations, we used chromHMM states inferred by the Roadmap Epigenomics Project (LCL: Gm12878 cell line, iPSC: H1hesc cell line, iPSC-CM: Right Ventricle) and caQTLs inferred previously. As before, we computed the proportion of eQTLs that are explained by each of the cell type-specific and shared genomic annotations (Supplementary Figure S19).

12 GTEx data

Two types of data were collected from the GTEx consortium (*Ardlie et al., 2015*). First we obtained summary statistics from the *cis* eQTL analysis. Specifically, for every gene tested in a tissue, the p-value of the lead variant was obtained. To overlap with eQTLs identified in iPSC-CMs the variant identified in the GTEx data was tested in iPSC-CMs. The QQ-plot was generated from a limited number of tissues for clarity (figs. S8). Raw RNA-seq fastq files were obtained (15 samples each) in Heart – Atrial Appendage,

Heart – Left Ventricle, Bone Marrow, Pancreas, Testis, Brain, Kidney, Colon, Lung, Skin, and Blood.

13 GWAS signal enrichments in gene expression data (Fig. 4A)

We used RolyPoly, a polygenic method that identifies trait-involved cell types by analyzing the enrichment of GWAS signal in cell type specific gene expression genome-wide. First, for each gene we calculate trait association scores by aggregating GWAS summary statistics from a window (10kb) centered on the TSS. Then, we estimate the individual contribution of each cell type to the observed gene score variance using a generalized linear regression model with normalized gene expression features. For each cell type we estimate an effect size coefficient and standard error, which we use for hypothesis testing. RolyPoly is available as an R package here `https://github.com/dcalderon/rolypoly`.

14 Stratified LDscore regression (Fig. 4B)

We obtained the LDscore regression software (*Bulik-Sullivan et al., 2015*) from `https://github.com/bulik/ldsc` and ran it using

```
python ldsc.py --h2 sumstats_file --ref-ld-chr annotation --w-ld-chr
eur_w_ld_chr --overlap-annot --frqfile-chr 1000G.mac5eur. --out outfile
--print-coefficient
```

To obtain heritability enrichment estimates, we ran stratified LDscore regression (*Finucane et al., 2015*) with each annotation separately plus annotations from the published baseline annotations, including:

Coding_UCSC, Coding_UCSC.extend.500, Conserved_LindbladToh, Conserved_LindbladToh.extend.500, Enhancer_Andersson, Enhancer_Andersson.extend.500, Enhancer_Hoffman, Enhancer_Hoffman.extend.500, Intron_UCSC, Intron_UCSC.extend.500, Promoter_UCSC, Promoter_UCSC.extend.500, SuperEnhancer_Hnisz, SuperEnhancer_Hnisz.extend.500, UTR_3_UCSC, UTR_3_UCSC.extend.500, UTR_5_UCSC, UTR_5_UCSC.extend.500, WeakEnhancer_Hoffman, and WeakEnhancer_Hoffman.extend.500

Summary statistics file were obtained from (*Okada et al., 2014*) (Rheumatoid Arthritis), (*Franke et al., 2010*) (Crohn's disease), the GIANT consortium (BMI), and CARDIoGRAMplusC4D (MI & CAD). To obtain a 95% confidence interval, we used $fc \pm 1.96s$, where fc and s are the LDscore fold enrichment estimates and standard error, respectively.

15 Neural network models for chromatin accessibility

Neural networks are highly expressive, nonlinear models that typically map a matrix representation of an input (here, DNA sequence of a genomic locus) to some output of interest (here, the chromatin accessibility state of that locus across cell types). Below, we describe in detail the different components of neural network models relevant to our problem of predicting chromatin accessibility, the model architectures of varying complexities used for our problem, our algorithm for learning the optimal model parameters, and key inferences made from our final model.

15.1 Model components

In deep neural networks, highly complex nonlinear mappings are typically constructed as a composition of several simpler nonlinear (or piece-wise linear) activation functions. Neural networks are typically represented by layers of variables (also called neurons) that connect an input layer of variables to an output layer of variables. A variable in each layer is a function of a linear combination of some subset of variables in the previous layer; the larger the number of layers, the deeper the neural network. The input layer consists of all the input variables in the model and the output layer consists of all the variables to be predicted.

Some commonly used intermediate layers are the dense layer, the convolutional layer, and the maxpool layer, and one commonly used activation function is the Rectified Linear Unit (ReLU).

In a dense layer with $\mathcal{J}$ variables, each variable depends on all the variables in the previous layer. Given a set of input variables $X_i, i \in \{1, \dots, \mathcal{I}\}$, each output variable Y_j of the dense layer can be defined as

$$Y_j = f\left(\sum_{i=1}^{\mathcal{I}} d_{ij} X_i\right) \tag{1}$$

A convolutional layer with $\mathcal{L}$ units is specified by $\mathcal{L}$ convolutional filters, each filter is represented by a matrix $\mathbf{C}^l_{mn}, m \in \{1, \dots, \mathcal{M}\}, n \in \{1, \dots, \mathcal{N}\}$. Given an input matrix $X_{ij}, i \in \{1, \dots, \mathcal{I}\}, j \in \{1, \dots, \mathcal{J}\}$, each filter maps the input matrix X to an output matrix Y^l of dimension $\mathcal{P} \times \mathcal{Q}$ where $\mathcal{P} = \mathcal{I} - \mathcal{M} + 1$ and $\mathcal{Q} = \mathcal{J} - \mathcal{N} + 1$.

$$Y^l_{pq} = f\left(\sum_{m,n} \mathbf{C}^l_{mn} X_{p+m,q+n}\right) \tag{2}$$

A maxpool layer with width w contains a single maxpool operation that is applied to contiguous, spatially ordered variables. Given a set of spatially ordered variables $X_i, i \in \{1, \dots, \mathcal{I}\}$, the maxpool operation gives

as output a new set of spatially ordered variables $Y_j, j \in \{1, \ldots, \frac{\mathcal{I}}{w}\}$ defined as

$$Y_j = \max\{X_{w\times j}, X_{w\times j+1}, \ldots, X_{w\times(j+1)}\} \tag{3}$$

The maxpool operation effectively smooths over local perturbations in a signal of interest. For example, if co-binding of two transcription factors occur as long as their high-affinity sequence elements lie within 10-15bp from each other, this co-binding signal can be detected more robustly by including a maxpool operation of width 5.

Finally, a common choice for $f(x)$ is the ReLU, defined as $f(x) = \max\{0, x\}$.

15.2 Model architectures

We are interested in predicting the chromatin activity of a genomic locus across three cell types (iPSC, LCL and iPSC-CM) from the DNA sequence of the locus alone. Thus, the input to the neural network model is a one-hot encoding of the reference DNA sequence of length 500bp centered at the locus, and the Input Layer consists of 4×500 binary-valued variables. The output of a neural network model is a categorical variable $O \in \{1, \ldots, 7\}$ where the values of the variables denote the following

$$O = \begin{cases} 1, & \text{if open in iPSC-CM alone} \\ 2, & \text{if open in LCL alone} \\ 3, & \text{if open in iPSC-CM and LCL} \\ 4, & \text{if open in iPSC alone} \\ 5, & \text{if open in iPSC and iPSC-CM} \\ 6, & \text{if open in iPSC and LCL} \\ 7, & \text{if open in all three cell types} \end{cases} \tag{4}$$

Since we are specifically interested in detecting sequence features that capture cell-specific and shared chromatin activity, we only include loci that are open in at least one cell type; this helps us ignore obvious sequence features (e.g., GC content) that distinguish open and closed chromatin. We used the sigmoid activation function to model the probability of the categorical variable in the output layer.

Fig. S16 illustrates several neural network models of varying depth and complexity (including OrbWeaver) that all use the same input and output layers. While the parameters of all the intermediate layers need to

be estimated for each of the models, the filters of the first convolutional layer in OrbWeaver were kept fixed to log-transformed position weight matrices (PWMs) of $1,320$ human transcription factors. For each TF, we used PWMs curated from two sources – TRANSFAC (*Matys et al., 2006*) and HT-SELEX (*Jolma et al., 2013*) – and, if a TF had more than one PWM, we selected the PWM with the highest information content. This approach is distinct from that taken in DanQ (*Quang and Xie, 2016*), where the filters in the first convolutional layer were initialized at known PWMs, but were still treated as free parameters to be estimated.

15.3 Model learning

We used a training set of $282,088$ loci to learn the parameters of each model using Adadelta (*Zeiler, 2012*), a stochastic gradient descent algorithm that adaptively computes learning rates. At each gradient descent step, the gradient is computed using a random batch of 1000 loci and optimization is terminated after one pass through the entire dataset. Fig. S17 compares the performance of each of the models on a test dataset of $7,151$ loci on chromosome 18. We computed the prediction for each cell type by collapsing the probability $p(O)$ for each of the 7 categories to marginal probabilities of chromatin activity.

$$\begin{aligned} p(\text{open in iPSC}) &= p(O=4)+p(O=5)+p(O=6)+p(O=7) \\ p(\text{open in LCL}) &= p(O=2)+p(O=3)+p(O=6)+p(O=7) \\ p(\text{open in iPSC-CM}) &= p(O=1)+p(O=3)+p(O=5)+p(O=7) \end{aligned} \tag{5}$$

We observed a diminishing increase in accuracy with increasing depth, and found that including known PWM information in the neural network helped increase the accuracy substantially. We haven't systematically explored a large space of deep neural networks, and acknowledge the possibility that a better designed, more complex architecture could outperform OrbWeaver. However, our analyses suggest that the gain in accuracy that could be achieved by deeper, more complex neural networks could more easily be achieved by incorporating known information about mechanisms underlying the output of interest (in this case, TF binding) into simpler neural networks.

15.4 Identifying key transcription factors for each category of chromatin activity

Fixing the filters in the first convolutional layer to known TF PWMs allowed us to directly query and interpret the importance of each of the factors in predicting active chromatin belonging to one of the 7 categories. We computed importance scores using DeepLIFT (*Shrikumar et al., 2016*), and for each of the 7 categories, we used loci belonging to that category if the model correctly predicted their category. For each locus, we calculated DeepLIFT scores on the input with respect to each filter in the first convolutional layer; this gives us a score for each TF at each position in the locus (i.e., a score matrix of size 1320×500). Note that these scores are different from those reported in the DeepLIFT paper, where DeepLIFT scores were typically computed with respect to the input layer rather than any intermediate layer. Each entry of the matrix quantifies the importance of the sequence affinity of a TF at a given position towards the category-specific prediction for that locus. For each locus, we designated the TF with the highest value in the score matrix as the TF that explains the chromatin activity state of that locus. For each category, we then computed a histogram across TFs of the proportion of loci explained by each TF. Table S7 lists the top 15 TFs for each category, and the proportion of sites explained by them.

15.5 Predicting effects of genetic variation on chromatin accessibility

We now set out to compare the observed (estimated) effects of genetic variants on chromatin accessibility at loci tested for caQTLs, and the effects of genetic variants predicted by OrbWeaver. First, for each cell type, we used qtlBHM, a Bayesian hierarchical model, without any annotation to compute the probability that a locus is a caQTL (π_l) and the probability that a SNP is the causal variant for a locus conditional on the locus being a caQTL (π_s). Restricting to loci with $\pi_l > 0.99$ and $\pi_s > 0.99$, using a 500 bp window centered at the causal variant of each such locus, we computed the OrbWeaver prediction at each of the 240 haplotypes (corresponding to 120 YRI individuals). Partitioning the haplotypes based on the alleles of the causal SNP, we then computed the difference in the median prediction of chromatin activity between the reference and alternate alleles for each of the three cell types. Fig. S18 compares this predicted allelic difference in chromatin activity with the observed allelic imbalance in ATAC-seq reads $\left(\frac{1}{2}\frac{\beta-\alpha}{\beta+\alpha}\right)$, computed using the α and β parameters output by WASP for that SNP-locus pair.

16 Raw and processed data availability

All data will be made available at `http://eqtl.uchicago.edu/Home.html`.

iPS-CM only (frac)	LCL only (frac)
**MEF2A (0.2359)** (***Ewen et al., 2011, Papait et al., 2013, Llucia-Valldeperas et al., 2014***)	**IRF1 (0.1917)** (***Sjostrand et al., 2016, Yamada et al., 1991***)
**TEAD4 (0.1835)** (***Benhaddou et al., 2012***)	IRF-6 (0.1598)
**GATA2 (0.1840)** (***Mauritz et al., 2008***)	**PU.1 (0.1470)** (***Heinz et al., 2010, Lin et al., 2010***)
TEAD3 (0.0392)	PEBP (0.0937)
GATA (0.0335)	RelB (0.0619)
NFIX (0.0387)	RPC155 (0.0426)
NFE2 (0.0624)	IRF (0.0381)
MRG2 (0.0395)	Oct-2 (0.0378)
ESRRB (0.0265)	NFKB2 (0.0263)
JunB (0.0234)	PAX5 (0.0258)
TFF-1 (0.0174)	PRDM1 (0.0175)
NF-1 (0.0120)	Six-5 (0.0108)
ZNF514 (0.0115)	IRF5 (0.0103)
meis1 (0.0082)	EBF1 (0.0102)
Hand1 (0.0061)	ZNF860 (0.0098)

iPS-CM+LCL (frac)	iPSC only (frac)
**Cap1 (0.1264)** (***Peche et al., 2013***)	**Oct3/4 (0.3850)** (***Nichols et al., 1998, Niwa et al., 2000***)
**c-Fos (0.1368)** (***Ohkubo et al., 2005***)	**Sox2/6 (0.1358)** (***Avilion et al., 2003***)
**FoxH1 (0.0768)** (***Bondue and Blanpain, 2010***)	**ZEB1 (0.0534)** (***Wellner et al., 2009***)
**MEF2A (0.0784)**	ZIC4 (0.0519)
**TEAD4 (0.0624)**	Rad21 (0.0509)
STAT5A (0.0440)	TEAD4 (0.0295)
PEBP (0.0368)	RPC155 (0.0163)
ZNF154 (0.0328)	Sp2 (0.0137)
**IRF1 (0.0296)**	MAZR (0.0137)
STAT4 (0.0288)	KLF14 (0.0137)
SPIB (0.0224)	RFX5 (0.0193)
MEF-2C (0.0216)	SMAD1 (0.0127)
Gata1 (0.0296)	INSM1 (0.0122)
TGIF (0.0168)	GRHL1 (0.0117)
MRG2 (0.0152)	SP4 (0.0112)

iPSC+iPS-CM (frac)	iPSC+LCL (frac)	iPSC+LCL+iPS-CM (frac)
**TEAD4 (0.4068)**	**Rad21 (0.2699)** (***Degner et al., 2009***)	**Rad21 (0.4480)** (***Degner et al., 2011***)
TEAD3 (0.1398)	**ZEB1 (0.1143)** (***Arnold et al., 2012***)	**CTCF (0.1177)** (***Degner et al., 2009, Balakrishnan et al., 2012***)
**Oct3/4 (0.0592)**	SPIB (0.0424)	LRF (0.0224)
**Sox2/6 (0.0416)**	EBF1 (0.0305)	Mad (0.0212)
Rad21 (0.0365)	PRDM6 (0.0261)	ESET (0.0178)
Zbtb5 (0.0189)	NFKB2 (0.0261)	BRCA1 (0.0173)
ZNF823 (0.0164)	RelB (0.0239)	CtBP1 (0.0157)
ZNF579 (0.0151)	Oct-2 (0.0218)	SP4 (0.0150)
**MEF2A (0.0151)**	ZNF480 (0.0196)	SMC-3 (0.0135)
c-MAF (0.0139)	**IRF1 (0.0185)**	sin3A (0.0132)
NFIX (0.0202)	SNAI2 (0.0163)	ZNF823 (0.0130)
Gsc (0.0113)	PAX5 (0.0163)	NF-E4 (0.0124)
NF-E4 (0.0101)	P50 (0.0163)	Rb (0.0122)
NF-AT3 (0.0101)	**Oct3/4 (0.0163)**	ZNF579 (0.0116)
Sp2 (0.0088)	PEBP (0.0152)	NRF1 (0.0104)

Table S7: Key transcription factors that are predictive of chromatin activity in shared and cell-specific open chromatin loci. For each TF, the number in parentheses denote the fraction of all loci in that category that have the TF PWM as the most important variable in the neural network, as quantified by DeepLIFT scores. Key factors that are known to be important for cell-specific and shared regulatory activity are shown in bold, with citations to previous work indicated alongside them.

Data	Accession
DNA methylation (LCL)	GSE57483 (GEO)
DNase-seq	GSE31388 (GEO)
RNA-seq (GEUVADIS)	E-GEUV-3 (ArrayExpress)
RNA-seq (iPSC, iPSC-CM)	GSE89895
ATAC-seq (iPSC, iPSC-CM)	GSE89895
DNA methylation (iPSC)	GSE89895

References

Ardlie et al., 2015. Ardlie, K. G., Deluca, D. S., Segre, A. V., Sullivan, T. J., Young, T. R., Gelfand, E. T., Trowbridge, C. A., Maller, J. B., Tukiainen, T., Lek, M., *et al.*, 2015. The Genotype-Tissue Expression

(GTEx) pilot analysis: multitissue gene regulation in humans. *Science*, **348**(6235):648–660.

Arnold et al., 2012. Arnold, C. N., Pirie, E., Dosenovic, P., McInerney, G. M., Xia, Y., Wang, N., Li, X., Siggs, O. M., Karlsson Hedestam, G. B., and Beutler, B., *et al.*, 2012. A forward genetic screen reveals roles for Nfkbid, Zeb1, and Ruvbl2 in humoral immunity. *Proc. Natl. Acad. Sci. U.S.A.*, **109**(31):12286–12293.

Avilion et al., 2003. Avilion, A. A., Nicolis, S. K., Pevny, L. H., Perez, L., Vivian, N., and Lovell-Badge, R., 2003. Multipotent cell lineages in early mouse development depend on SOX2 function. *Genes Dev.*, **17**(1):126–140.

Balakrishnan et al., 2012. Balakrishnan, S. K., Witcher, M., Berggren, T. W., and Emerson, B. M., 2012. Functional and molecular characterization of the role of CTCF in human embryonic stem cell biology. *PLoS ONE*, **7**(8):e42424.

Banovich et al., 2014. Banovich, N. E., Lan, X., McVicker, G., van de Geijn, B., Degner, J. F., Blischak, J. D., Roux, J., Pritchard, J. K., and Gilad, Y., 2014. Methylation QTLs are associated with coordinated changes in transcription factor binding, histone modifications, and gene expression levels. *PLoS Genet.*, **10**(9):e1004663.

Benhaddou et al., 2012. Benhaddou, A., Keime, C., Ye, T., Morlon, A., Michel, I., Jost, B., Mengus, G., and Davidson, I., 2012. Transcription factor TEAD4 regulates expression of myogenin and the unfolded protein response genes during C2C12 cell differentiation. *Cell Death Differ.*, **19**(2):220–231.

Bondue and Blanpain, 2010. Bondue, A. and Blanpain, C., 2010. Mesp1: a key regulator of cardiovascular lineage commitment. *Circ. Res.*, **107**(12):1414–1427.

Bray et al., 2016. Bray, N. L., Pimentel, H., Melsted, P., and Pachter, L., 2016. Near-optimal probabilistic RNA-seq quantification. *Nat. Biotechnol.*, **34**(5):525–527.

Buenrostro et al., 2013. Buenrostro, J. D., Giresi, P. G., Zaba, L. C., Chang, H. Y., and Greenleaf, W. J., 2013. Transposition of native chromatin for fast and sensitive epigenomic profiling of open chromatin, DNA-binding proteins and nucleosome position. *Nat. Methods*, **10**(12):1213–1218.

Bulik-Sullivan et al., 2015. Bulik-Sullivan, B. K., Loh, P. R., Finucane, H. K., Ripke, S., Yang, J., Patterson, N., Daly, M. J., Price, A. L., Neale, B. M., Ripke, S., *et al.*, 2015. LD Score regression distinguishes confounding from polygenicity in genome-wide association studies. *Nat. Genet.*, **47**(3):291–295.

Burridge et al., 2014. Burridge, P. W., Matsa, E., Shukla, P., Lin, Z. C., Churko, J. M., Ebert, A. D., Lan, F., Diecke, S., Huber, B., Mordwinkin, N. M., *et al.*, 2014. Chemically defined generation of human cardiomyocytes. *Nat. Methods*, **11**(8):855–860.

Burrows et al., 2016. Burrows, C. K., Banovich, N. E., Pavlovic, B. J., Patterson, K., Gallego Romero, I., Pritchard, J. K., and Gilad, Y., 2016. Genetic Variation, Not Cell Type of Origin, Underlies the Majority of Identifiable Regulatory Differences in iPSCs. *PLoS Genet.*, **12**(1):e1005793.

Chan et al., 2013. Chan, Y. C., Ting, S., Lee, Y. K., Ng, K. M., Zhang, J., Chen, Z., Siu, C. W., Oh, S. K., and Tse, H. F., 2013. Electrical stimulation promotes maturation of cardiomyocytes derived from human embryonic stem cells. *J Cardiovasc Transl Res*, **6**(6):989–999.

Degner et al., 2012. Degner, J. F., Pai, A. A., Pique-Regi, R., Veyrieras, J. B., Gaffney, D. J., Pickrell, J. K., De Leon, S., Michelini, K., Lewellen, N., Crawford, G. E., *et al.*, 2012. DNase I sensitivity QTLs are a major determinant of human expression variation. *Nature*, **482**(7385):390–394.

Degner et al., 2011. Degner, S. C., Verma-Gaur, J., Wong, T. P., Bossen, C., Iverson, G. M., Torkamani, A., Vettermann, C., Lin, Y. C., Ju, Z., Schulz, D., *et al.*, 2011. CCCTC-binding factor (CTCF) and cohesin influence the genomic architecture of the Igh locus and antisense transcription in pro-B cells. *Proc. Natl. Acad. Sci. U.S.A.*, **108**(23):9566–9571.

Degner et al., 2009. Degner, S. C., Wong, T. P., Jankevicius, G., and Feeney, A. J., 2009. Cutting edge: developmental stage-specific recruitment of cohesin to CTCF sites throughout immunoglobulin loci during B lymphocyte development. *J. Immunol.*, **182**(1):44–48.

Dobin et al., 2013. Dobin, A., Davis, C. A., Schlesinger, F., Drenkow, J., Zaleski, C., Jha, S., Batut, P., Chaisson, M., and Gingeras, T. R., 2013. STAR: ultrafast universal RNA-seq aligner. *Bioinformatics*, **29**(1):15–21.

Dunham et al., 2012. Dunham, I., Kundaje, A., Aldred, S. F., Collins, P. J., Davis, C. A., Doyle, F., Epstein, C. B., Frietze, S., Harrow, J., Kaul, R., *et al.*, 2012. An integrated encyclopedia of DNA elements in the human genome. *Nature*, **489**(7414):57–74.

Ewen et al., 2011. Ewen, E. P., Snyder, C. M., Wilson, M., Desjardins, D., and Naya, F. J., 2011. The Mef2A transcription factor coordinately regulates a costamere gene program in cardiac muscle. *J. Biol. Chem.*, **286**(34):29644–29653.

Finucane et al., 2015. Finucane, H. K., Bulik-Sullivan, B., Gusev, A., Trynka, G., Reshef, Y., Loh, P. R., Anttila, V., Xu, H., Zang, C., Farh, K., *et al.*, 2015. Partitioning heritability by functional annotation using genome-wide association summary statistics. *Nat. Genet.*, **47**(11):1228–1235.

Franke et al., 2010. Franke, A., McGovern, D. P., Barrett, J. C., Wang, K., Radford-Smith, G. L., Ahmad, T., Lees, C. W., Balschun, T., Lee, J., Roberts, R., *et al.*, 2010. Genome-wide meta-analysis increases to 71 the number of confirmed Crohn's disease susceptibility loci. *Nat. Genet.*, **42**(12):1118–1125.

Heinz et al., 2010. Heinz, S., Benner, C., Spann, N., Bertolino, E., Lin, Y. C., Laslo, P., Cheng, J. X., Murre, C., Singh, H., and Glass, C. K., *et al.*, 2010. Simple combinations of lineage-determining transcription factors prime cis-regulatory elements required for macrophage and B cell identities. *Mol. Cell*, **38**(4):576–589.

Jolma et al., 2013. Jolma, A., Yan, J., Whitington, T., Toivonen, J., Nitta, K. R., Rastas, P., Morgunova, E., Enge, M., Taipale, M., Wei, G., *et al.*, 2013. DNA-binding specificities of human transcription factors. *Cell*, **152**(1-2):327–339.

Lappalainen et al., 2013. Lappalainen, T., Sammeth, M., Friedlander, M. R., 't Hoen, P. A., Monlong, J., Rivas, M. A., Gonzalez-Porta, M., Kurbatova, N., Griebel, T., Ferreira, P. G., *et al.*, 2013. Transcriptome and genome sequencing uncovers functional variation in humans. *Nature*, **501**(7468):506–511.

Li et al., 2016. Li, Y. I., van de Geijn, B., Raj, A., Knowles, D. A., Petti, A. A., Golan, D., Gilad, Y., and Pritchard, J. K., 2016. RNA splicing is a primary link between genetic variation and disease. *Science*, **352**(6285):600–604.

Lian et al., 2013. Lian, X., Zhang, J., Azarin, S. M., Zhu, K., Hazeltine, L. B., Bao, X., Hsiao, C., Kamp, T. J., and Palecek, S. P., 2013. Directed cardiomyocyte differentiation from human pluripotent stem cells by modulating Wnt/β-catenin signaling under fully defined conditions. *Nat Protoc*, **8**(1):162–175.

Lin et al., 2010. Lin, Y. C., Jhunjhunwala, S., Benner, C., Heinz, S., Welinder, E., Mansson, R., Sigvardsson, M., Hagman, J., Espinoza, C. A., Dutkowski, J., *et al.*, 2010. A global network of transcription factors, involving E2A, EBF1 and Foxo1, that orchestrates B cell fate. *Nat. Immunol.*, **11**(7):635–643.

Llucia-Valldeperas et al., 2014. Llucia-Valldeperas, A., Sanchez, B., Soler-Botija, C., Galvez-Monton, C., Roura, S., Prat-Vidal, C., Perea-Gil, I., Rosell-Ferrer, J., Bragos, R., and Bayes-Genis, A., *et al.*, 2014. Physiological conditioning by electric field stimulation promotes cardiomyogenic gene expression in human cardiomyocyte progenitor cells. *Stem Cell Res Ther*, **5**(4):93.

Marroquin et al., 2007. Marroquin, L. D., Hynes, J., Dykens, J. A., Jamieson, J. D., and Will, Y., 2007. Circumventing the Crabtree effect: replacing media glucose with galactose increases susceptibility of HepG2 cells to mitochondrial toxicants. *Toxicol. Sci.*, **97**(2):539–547.

Matys et al., 2006. Matys, V., Kel-Margoulis, O. V., Fricke, E., Liebich, I., Land, S., Barre-Dirrie, A., Reuter, I., Chekmenev, D., Krull, M., Hornischer, K., *et al.*, 2006. TRANSFAC and its module TRANSCompel: transcriptional gene regulation in eukaryotes. *Nucleic Acids Res.*, **34**(Database issue):D108–110.

Mauritz et al., 2008. Mauritz, C., Schwanke, K., Reppel, M., Neef, S., Katsirntaki, K., Maier, L. S., Nguemo, F., Menke, S., Haustein, M., Hescheler, J., *et al.*, 2008. Generation of functional murine cardiac myocytes from induced pluripotent stem cells. *Circulation*, **118**(5):507–517.

Muller et al., 2011. Muller, F. J., Schuldt, B. M., Williams, R., Mason, D., Altun, G., Papapetrou, E. P., Danner, S., Goldmann, J. E., Herbst, A., Schmidt, N. O., *et al.*, 2011. A bioinformatic assay for pluripotency in human cells. *Nat. Methods*, **8**(4):315–317.

Nichols et al., 1998. Nichols, J., Zevnik, B., Anastassiadis, K., Niwa, H., Klewe-Nebenius, D., Chambers, I., Scholer, H., and Smith, A., 1998. Formation of pluripotent stem cells in the mammalian embryo depends on the POU transcription factor Oct4. *Cell*, **95**(3):379–391.

Niwa et al., 2000. Niwa, H., Miyazaki, J., and Smith, A. G., 2000. Quantitative expression of Oct-3/4 defines differentiation, dedifferentiation or self-renewal of ES cells. *Nat. Genet.*, **24**(4):372–376.

Ohkubo et al., 2005. Ohkubo, Y., Arima, M., Arguni, E., Okada, S., Yamashita, K., Asari, S., Obata, S., Sakamoto, A., Hatano, M., O-Wang, J., *et al.*, 2005. A role for c-fos/activator protein 1 in B lymphocyte terminal differentiation. *J. Immunol.*, **174**(12):7703–7710.

Okada et al., 2014. Okada, Y., Wu, D., Trynka, G., Raj, T., Terao, C., Ikari, K., Kochi, Y., Ohmura, K., Suzuki, A., Yoshida, S., *et al.*, 2014. Genetics of rheumatoid arthritis contributes to biology and drug discovery. *Nature*, **506**(7488):376–381.

Okita et al., 2011. Okita, K., Matsumura, Y., Sato, Y., Okada, A., Morizane, A., Okamoto, S., Hong, H., Nakagawa, M., Tanabe, K., Tezuka, K., *et al.*, 2011. A more efficient method to generate integration-free human iPS cells. *Nat. Methods*, **8**(5):409–412.

Ongen et al., 2016. Ongen, H., Buil, A., Brown, A. A., Dermitzakis, E. T., and Delaneau, O., 2016. Fast and efficient QTL mapper for thousands of molecular phenotypes. *Bioinformatics*, **32**(10):1479–1485.

Papait et al., 2013. Papait, R., Cattaneo, P., Kunderfranco, P., Greco, C., Carullo, P., Guffanti, A., Vigano, V., Stirparo, G. G., Latronico, M. V., Hasenfuss, G., *et al.*, 2013. Genome-wide analysis of histone marks identifying an epigenetic signature of promoters and enhancers underlying cardiac hypertrophy. *Proc. Natl. Acad. Sci. U.S.A.*, **110**(50):20164–20169.

Peche et al., 2013. Peche, V. S., Holak, T. A., Burgute, B. D., Kosmas, K., Kale, S. P., Wunderlich, F. T., Elhamine, F., Stehle, R., Pfitzer, G., Nohroudi, K., *et al.*, 2013. Ablation of cyclase-associated protein 2 (CAP2) leads to cardiomyopathy. *Cell. Mol. Life Sci.*, **70**(3):527–543.

Pique-Regi et al., 2011. Pique-Regi, R., Degner, J. F., Pai, A. A., Gaffney, D. J., Gilad, Y., and Pritchard, J. K., 2011. Accurate inference of transcription factor binding from DNA sequence and chromatin accessibility data. *Genome Res.*, **21**(3):447–455.

Quang and Xie, 2016. Quang, D. and Xie, X., 2016. DanQ: a hybrid convolutional and recurrent deep neural network for quantifying the function of DNA sequences. *Nucleic Acids Res.*, **44**(11):e107.

Shrikumar et al., 2016. Shrikumar, A., Greenside, P., Shcherbina, A., and Kundaje, A., 2016. Not just a black box: Learning important features through propagating activation differences. *CoRR*, **abs/1605.01713**.

Sjostrand et al., 2016. Sjostrand, M., Johansson, A., Aqrawi, L., Olsson, T., Wahren-Herlenius, M., and Espinosa, A., 2016. The Expression of BAFF Is Controlled by IRF Transcription Factors. *J. Immunol.*, **196**(1):91–96.

Storey and Tibshirani, 2003. Storey, J. D. and Tibshirani, R., 2003. Statistical significance for genomewide studies. *Proc. Natl. Acad. Sci. U.S.A.*, **100**(16):9440–9445.

Tohyama et al., 2013. Tohyama, S., Hattori, F., Sano, M., Hishiki, T., Nagahata, Y., Matsuura, T., Hashimoto, H., Suzuki, T., Yamashita, H., Satoh, Y., *et al.*, 2013. Distinct metabolic flow enables large-scale purification of mouse and human pluripotent stem cell-derived cardiomyocytes. *Cell Stem Cell*, **12**(1):127–137.

van de Geijn et al., 2015. van de Geijn, B., McVicker, G., Gilad, Y., and Pritchard, J. K., 2015. WASP: allele-specific software for robust molecular quantitative trait locus discovery. *Nat. Methods*, **12**(11):1061–1063.

Wang et al., 2014. Wang, G., McCain, M. L., Yang, L., He, A., Pasqualini, F. S., Agarwal, A., Yuan, H., Jiang, D., Zhang, D., Zangi, L., *et al.*, 2014. Modeling the mitochondrial cardiomyopathy of Barth syndrome with induced pluripotent stem cell and heart-on-chip technologies. *Nat. Med.*, **20**(6):616–623.

Wellner et al., 2009. Wellner, U., Schubert, J., Burk, U. C., Schmalhofer, O., Zhu, F., Sonntag, A., Waldvogel, B., Vannier, C., Darling, D., zur Hausen, A., *et al.*, 2009. The EMT-activator ZEB1 promotes tumorigenicity by repressing stemness-inhibiting microRNAs. *Nat. Cell Biol.*, **11**(12):1487–1495.

Yamada et al., 1991. Yamada, G., Ogawa, M., Akagi, K., Miyamoto, H., Nakano, N., Itoh, S., Miyazaki, J., Nishikawa, S., Yamamura, K., and Taniguchi, T., *et al.*, 1991. Specific depletion of the B-cell population induced by aberrant expression of human interferon regulatory factor 1 gene in transgenic mice. *Proc. Natl. Acad. Sci. U.S.A.*, **88**(2):532–536.

Zeiler, 2012. Zeiler, M. D., 2012. ADADELTA: an adaptive learning rate method. *CoRR*, **abs/1212.5701**.

Supplementary Figures

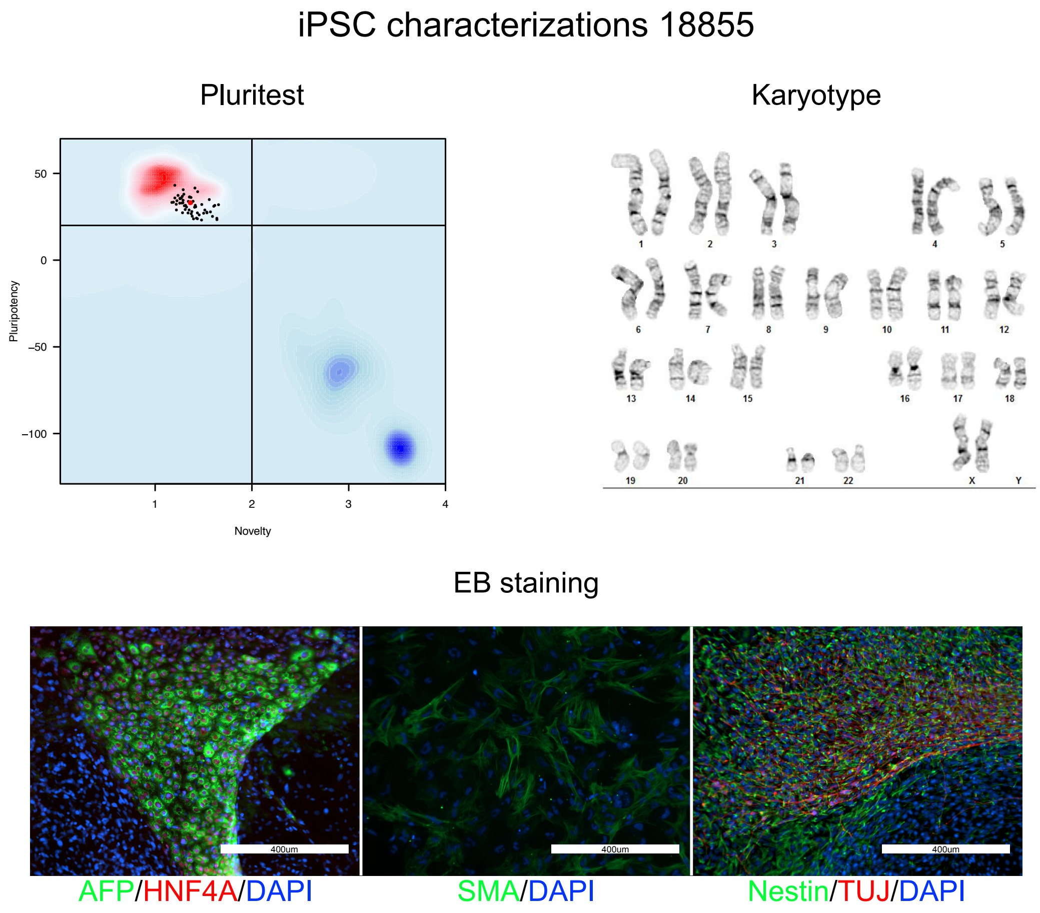

Figure S1: Pluritest results were plotted for each individual. The red dot corresponds to the NA18855 sample. Black lines represent the empirical quality thresholds. Karyotype results are shown for NA18855. Immunostaining performed on spontaneously differentiated tissue derived from embryoid bodies (EBs) confirms cells types from all three germ layers - endoderm (AFP/HNF4A), mesoderm (SMA), and ectoderm (Nestin/TUJ/Map2). QC for other lines can be found at `http://eqtl.uchicago.edu/yri_ipsc/iPSC_QC.pdf`.

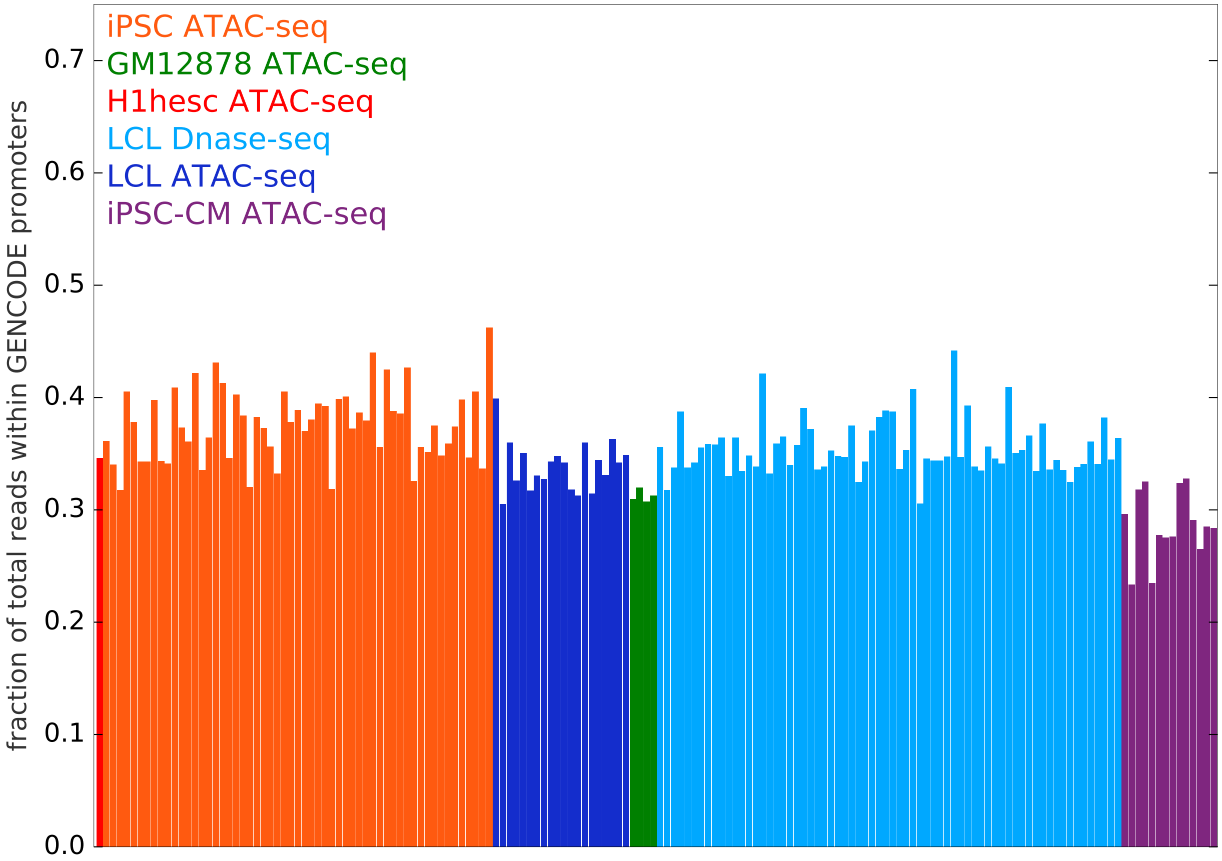

Figure S2: **Quantifying library quality using proportion of reads in promoters.** The proportion of ATAC-seq reads within promoters in YRI LCLs is comparable to the proportion of ATAC-seq reads in GM12878 (*Buenrostro et al., 2013*) and the proportion of DNase-seq reads within promoters in the same group of YRI LCLs (*Degner et al., 2012*). The proportion of ATAC-seq reads for iPSCs is also comparable to the proportion of ATAC-seq reads for H1hesc (courtesy of the Greenleaf Lab). The proportion of ATAC-seq reads for iPSC-CMs is slightly lower compared to other cell types indicating that a larger fraction of the transpositions in these libraries occur within distal enhancers.

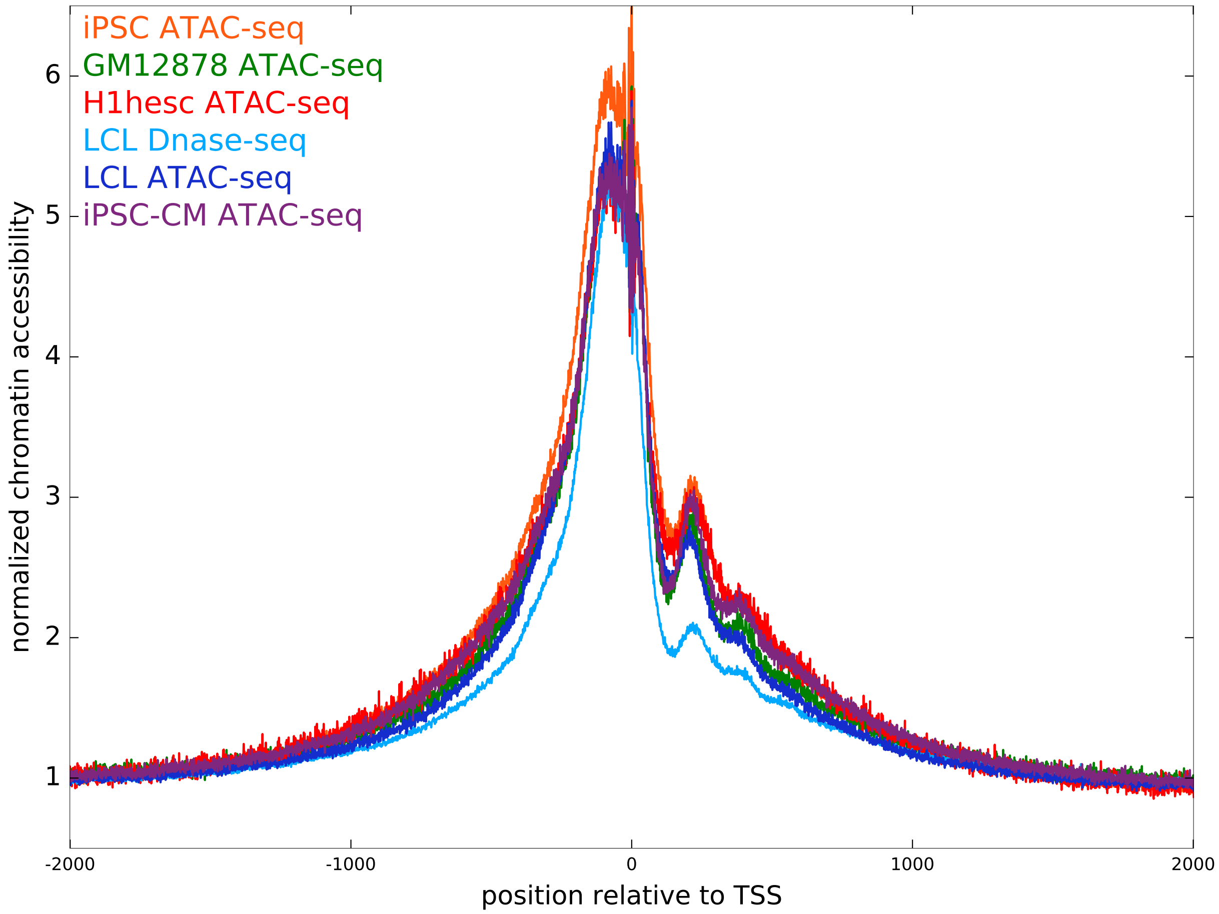

Figure S3: **Quantifying library quality using chromatin accessibility around TSS.** Chromatin accessibility in each cell type is computed as the count of Dnase nicks or ATAC transpositions centered at the transcription start site, normalized by the accessibility signal 2kb upstream of the TSS, mean-averaged across all GENCODE genes and median-averaged across multiple individuals or libraries. The accessibility profile across all ATAC-seq libraries are highly similar to each other and slightly broader than the accessibility profile of DNase-seq libraries.

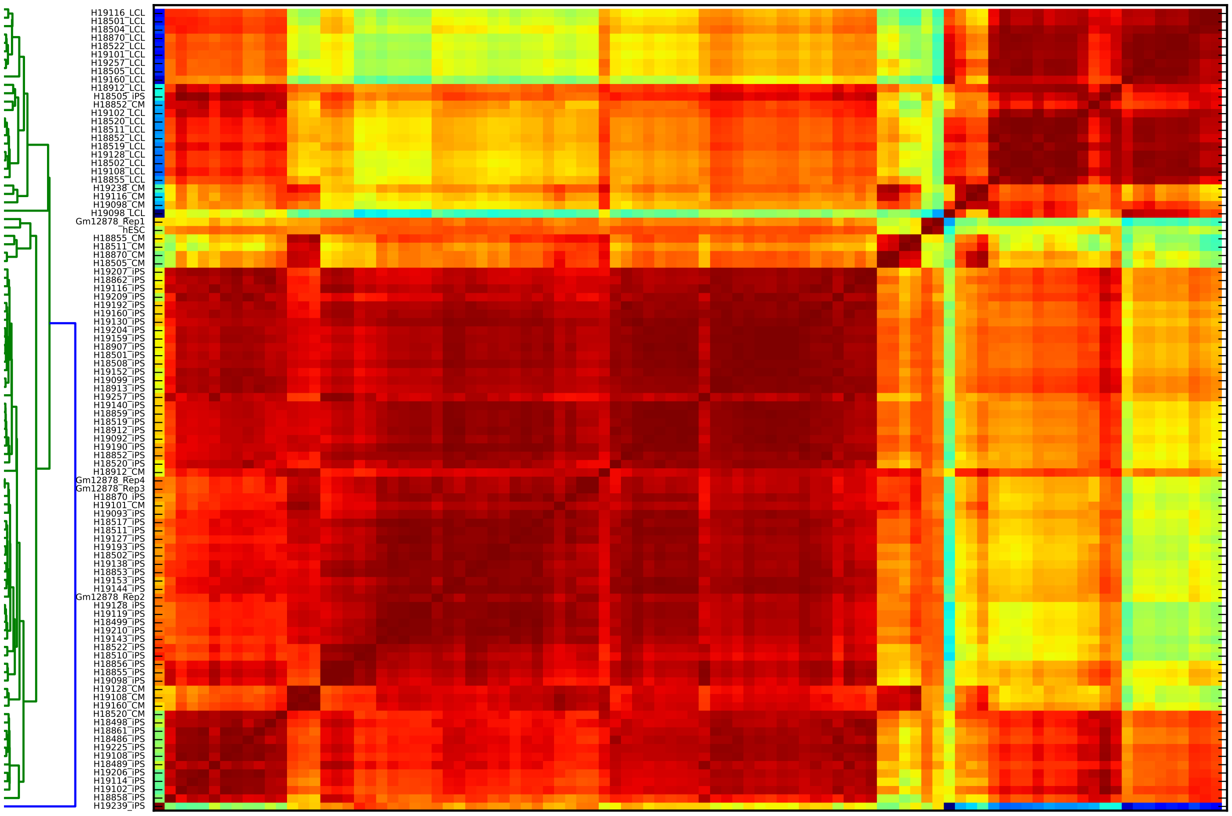

Figure S4: **Comparing the fragment-size distributions between ATAC-seq libraries.** A heatmap illustrating the correlation of fragment-size distributions between several ATAC-seq libraries of LCLs, iPSCs, and iPSC-CMs, along with previously published data for LCL (*Buenrostro et al., 2013*) and H1hesc (courtesy of the Greenleaf Lab). In general, libraries for each cell type cluster together, with the exception of a few iPSC-CM libraries. All iPSC and iPSC-CM libraries had very similar characteristics, with a substantial fraction of fragments spanning one or two nucleosomes. The LCL libraries were subtly different, with a much smaller fraction of fragments spanning nucleosomes.

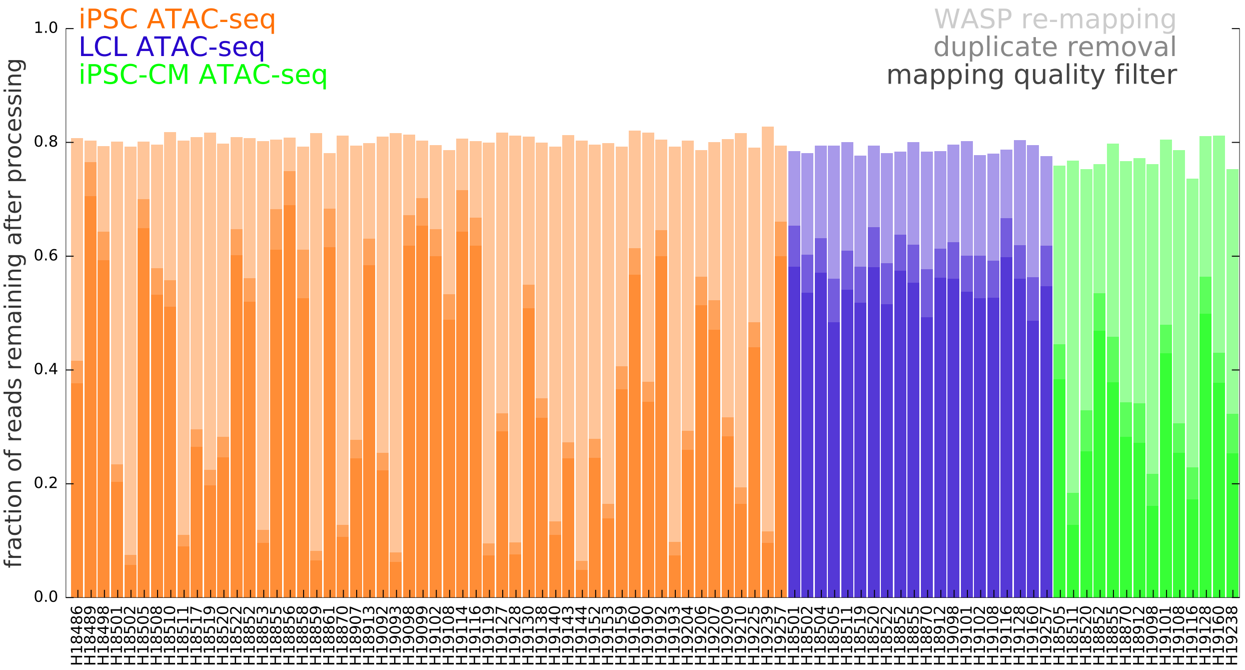

Figure S5: **Quantifying ATAC-seq read loss at various data processing steps.** At each step of processing the ATAC-seq data, the fraction of nuclear DNA reads filtered out varied across cell types and individuals. The fraction of reads filtered out by WASP remapping was relatively stable at ≈ 20%, as was the fraction removed using a mapping quality filter of 10 (≈ 5%). The fraction of reads removed due to filtering out duplicate fragments varied substantially across libraries.

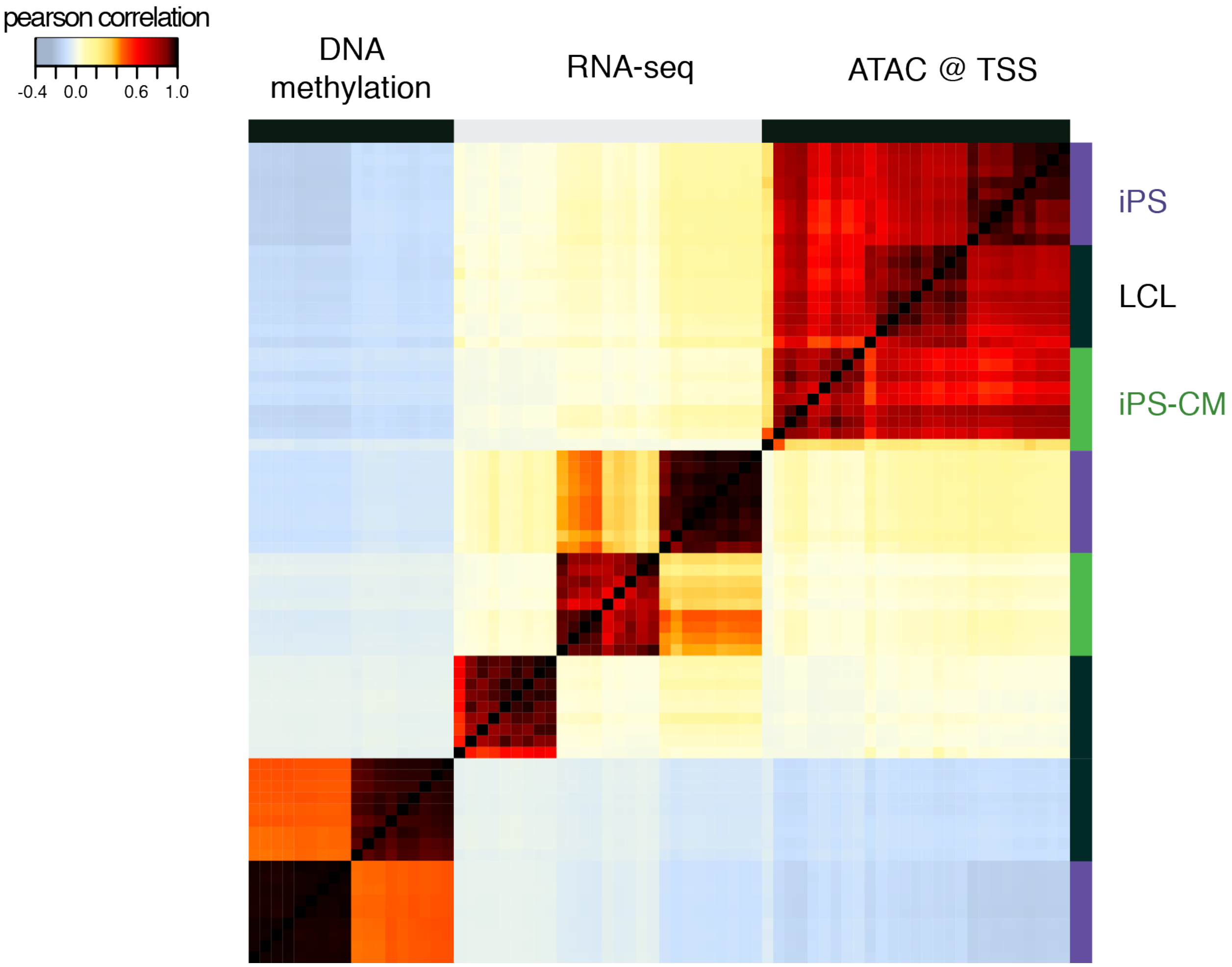

Figure S6: Correlation of measurements across molecular phenotype, individuals, and cell types. Individuals were chosen at random among the iPSC-CM samples. Genic ATAC-seq and DNA methylation signal were measured at the TSS (±1kbp) and at a randomly chosen methylation probe overlapping the gene body, respectively. The correlation structure is consistent with highly reproducible molecular measurements between biological replicates, but also matches our expectations that, at the global scale, chromatin accessibility and RNA expression measurements should be correlated while DNA methylation should be anti-correlated with RNA expression and chromatin accessibility.

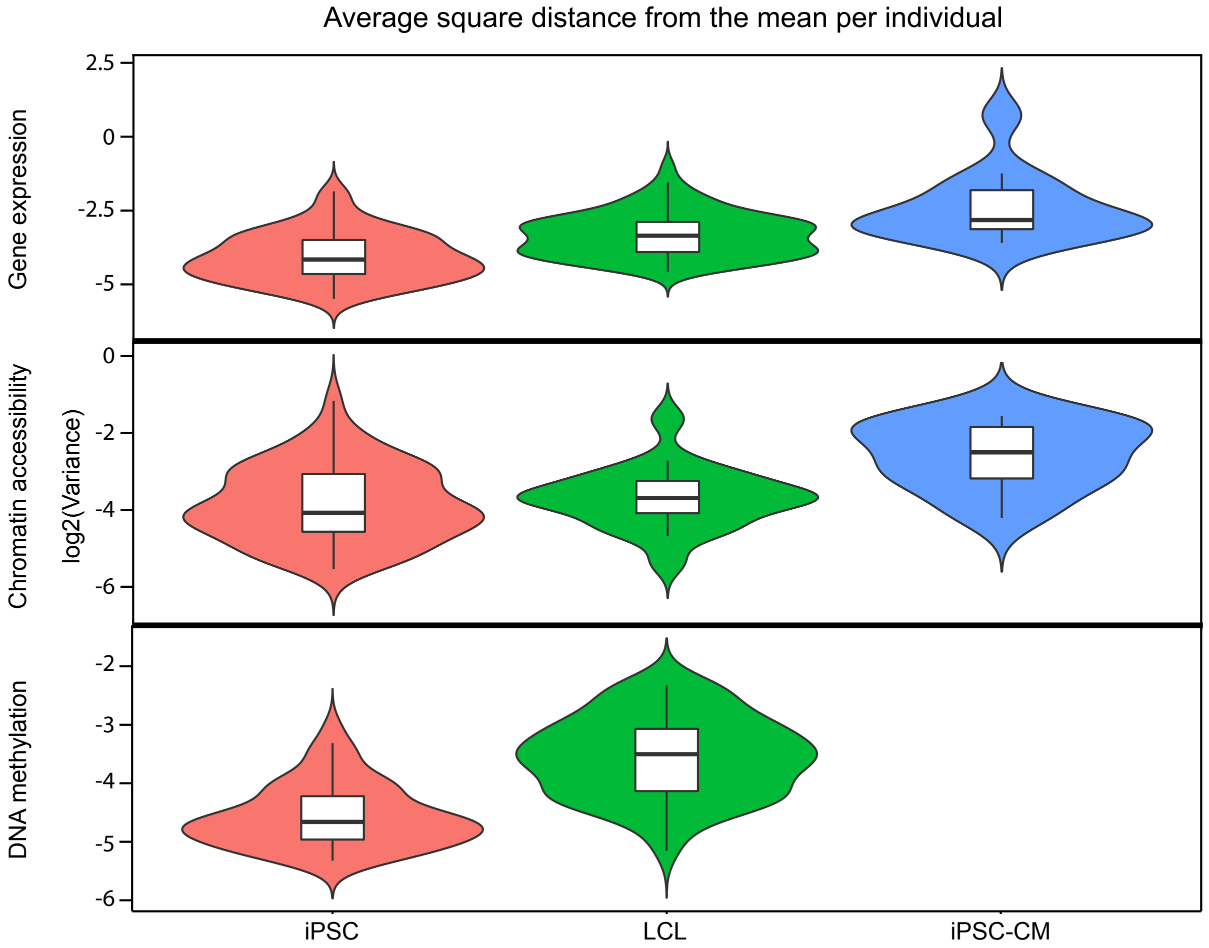

Figure S7: Violin plots representing per individual $\log_2$ of the average square distance from the mean for iPSC, LCL, and iPSC-CM gene expression, chromatin accessibility, and DNA methylation levels (methylation not assayed in iPSC-CMs).

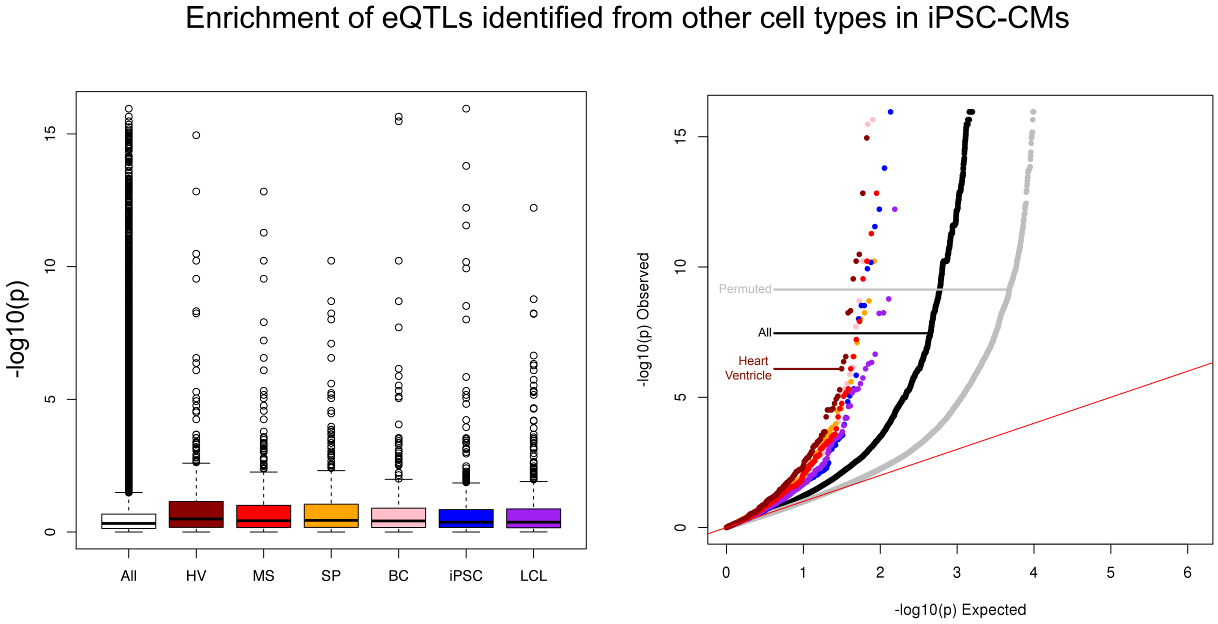

Figure S8: Boxplots of the $-\log_{10}$ p-value from eQTLs identified in iPSC-CMs conditioned on being previously identified as eQTLs in tissues from the GTEx consortium as well as iPSCs and LCLs. Specifically, the GTEx tissues heart left ventricle (HV), skeletal muscle (MS), spleen (SP), and the cortex of the brain (BC) were used. eQTLs identified in heart left ventricle show the greatest enrichment of associations with expression in iPSC-CMs.

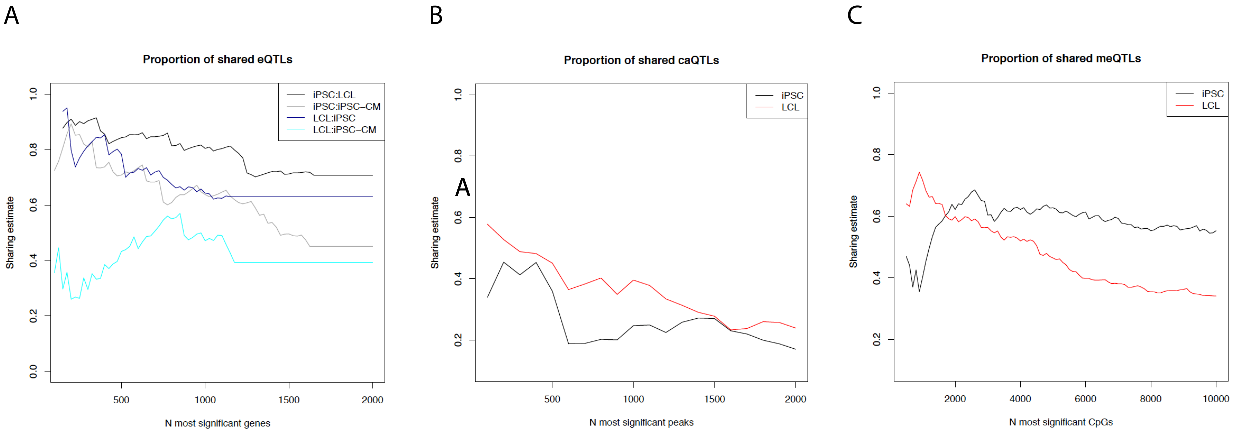

Figure S9: π_1 **estimates of sharing** of expression QTLs (A), chromatin accessibility QTLs (B), and DNA methylation QTLs (B) estimated across the top N most significant genes, peaks, and probes, respectively.

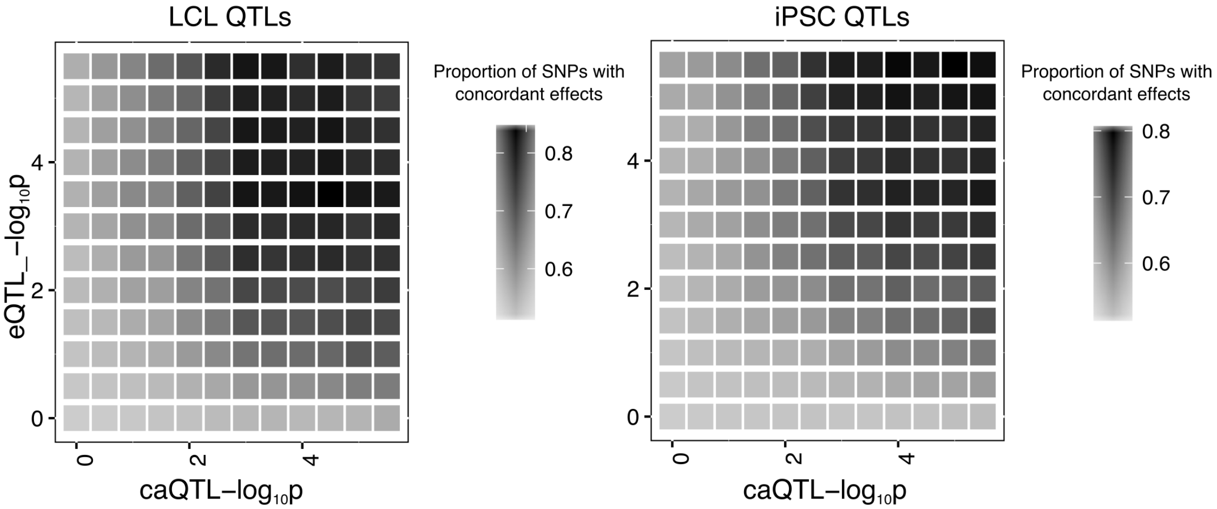

Figure S10: Concordance of effect size directions between eQTLs and caQTLs identified in LCLs (left panel) and IPS (right panel). At the strongest level of significance ($-\log_{10} p > 5$ for both eQTL and caQTL), we find that for over 80% of SNPs, effects on chromatin accessibility and gene expression are positively correlated.

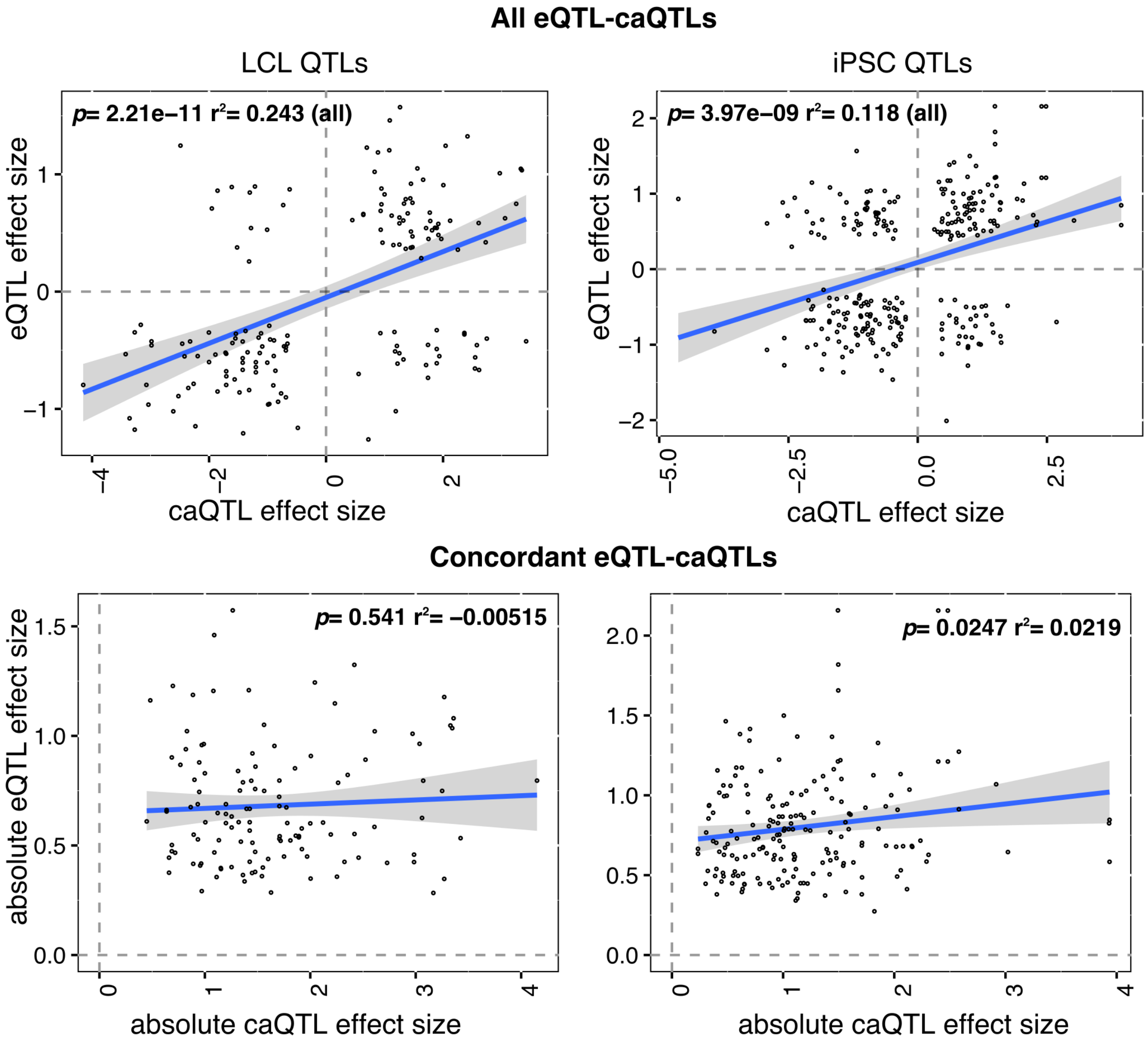

Figure S11: Top panel: Comparison between caQTL and eQTL effect sizes for QTLs identified in LCLs and IPS. Weak predictive power (linear regression) of caQTL effect sizes for eQTL effect sizes. Bottom panel: magnitudes of caQTL effect sizes show no predict power for the magnitudes of eQTL effects. The caQTL-eQTL pairs with discordant directions of effect were removed from this analysis.

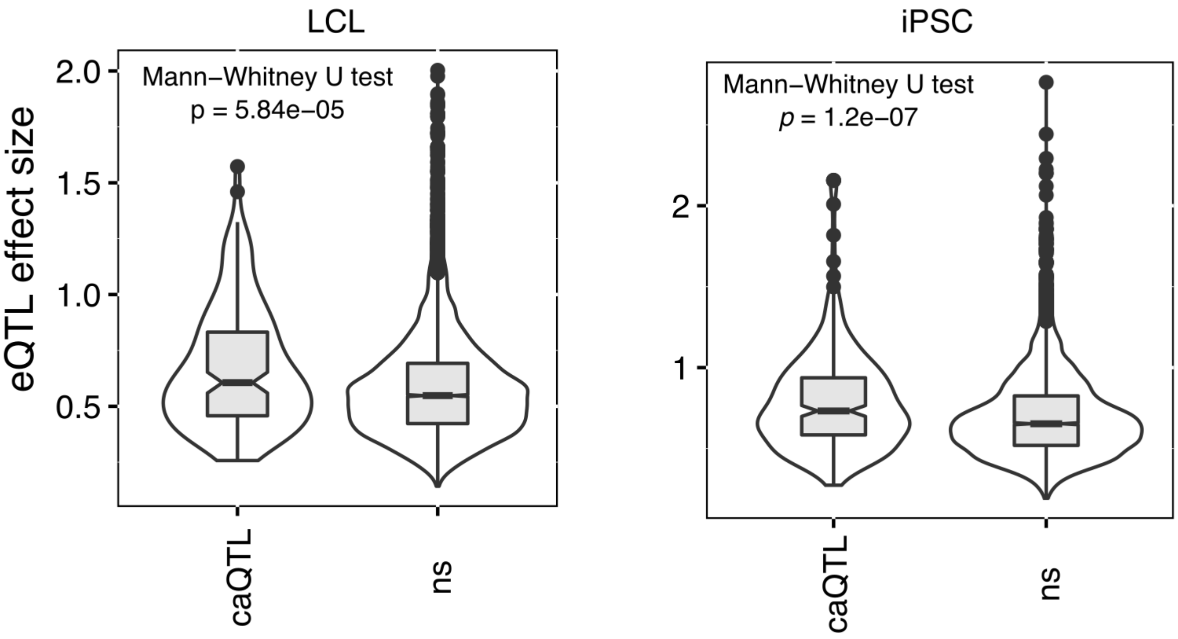

Figure S12: Comparison of absolute effect sizes between eQTLs that have an associated caQTLs and those that do not. In both LCLs and iPSCs, eQTLs that are caQTLs show larger effect sizes than compared to those with no caQTLs.

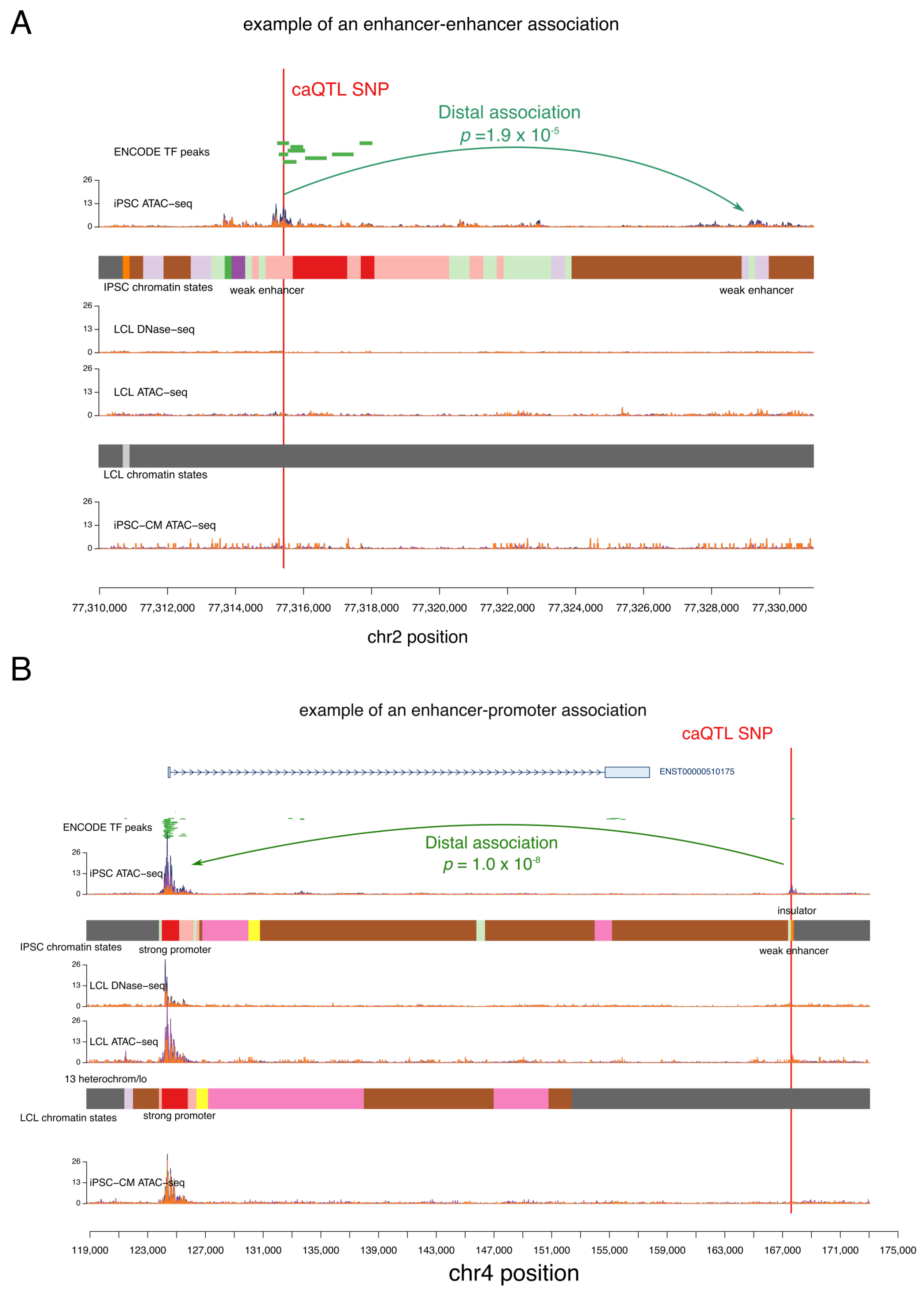

Figure S13: **Examples of distal caQTLs affecting a chromatin element interactions.** **(A)** Example of an iPSC-specific caQTL SNP located in a weak enhancer in iPSC and in LCL heterochromatin. This SNP affects accessibility at a distal enhancer element in iPSCs, but not in LCLs. **(B)** Example of another iPSC-specific caQTL SNP located in an iPSC enhancer and in LCL heterochromatin. This SNP has an iPSC-specific distal effect at a promoter that is active in all three cell types (iPSC, LCLs, and iPSC-CM)

A

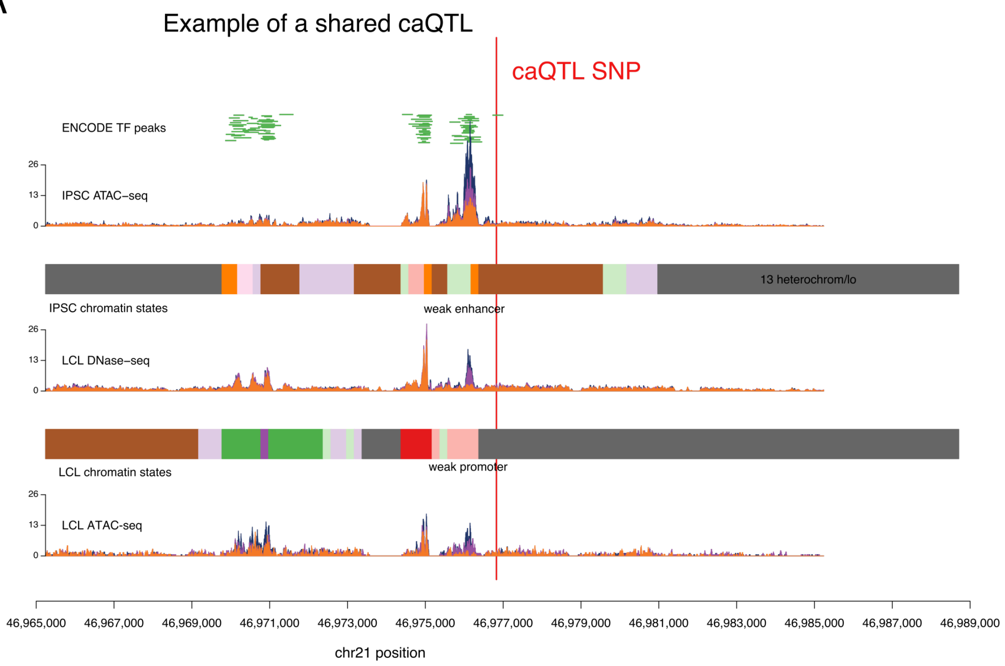

B

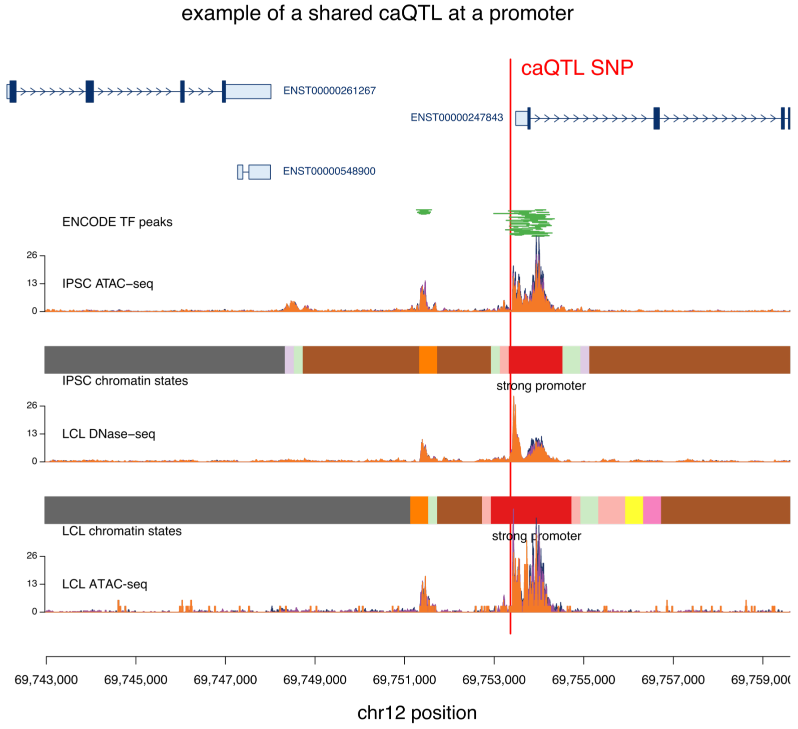

Figure S14: **Examples of shared caQTLs in LCLs and IPSCs.** **(A)** Example of a shared caQTL in an intergenic region. The associated caQTL SNP is located near a shared accessibility peak, and overlaps a predicted transcription factor binding site from ENCODE. The QTL effect is seen in both LCL ATAC-seq and DNase-seq data. **(B)** Example of a shared caQTL in a strong promoter region as determined by chromHMM predictions.

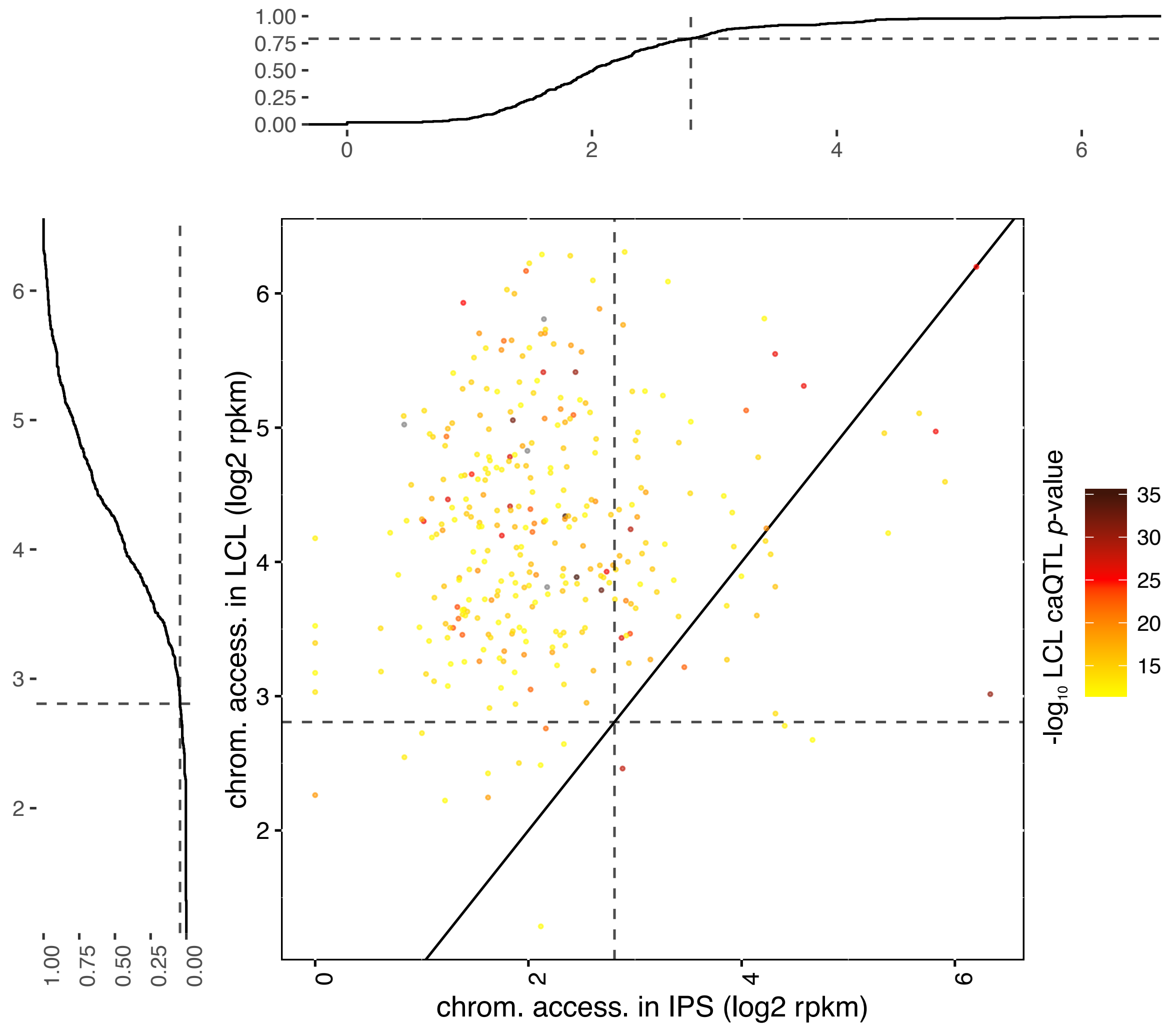

Figure S15: **LCL-specific caQTLs chromatin accessibility signal in LCLs and iPSCs.** Cumulative distributions of LCL-specific caQTL chromatin accessibility in LCL and iPSC show that most LCL-specific caQTLs (> 90%) are located in accessible regions in LCLs but only about 20% are located in iPSC-accessible regions.

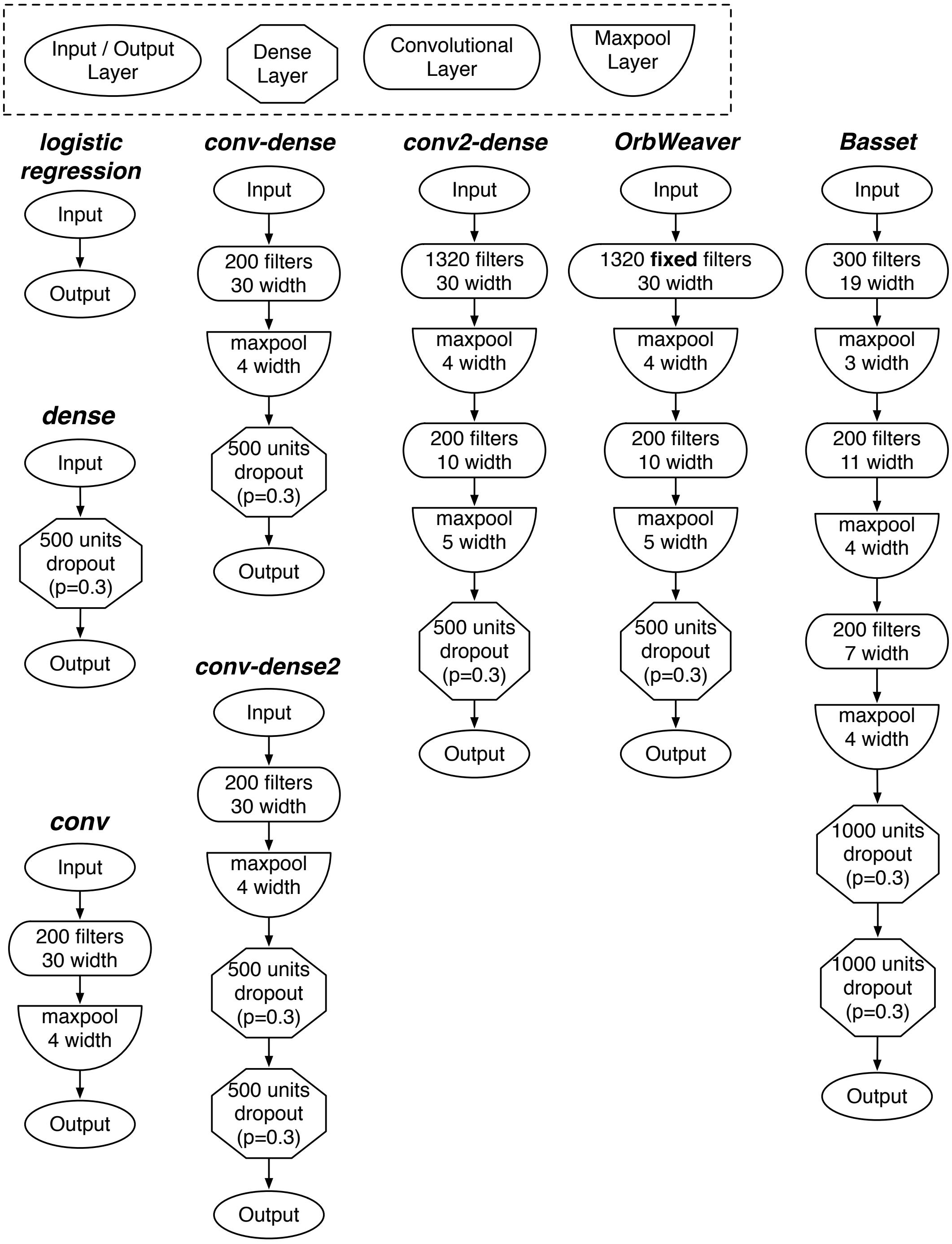

Figure S16: **Deep neural network architectures for predicting chromatin activity.** We illustrate deep neural network models of increasing complexity, including OrbWeaver and Basset, each of which are built by layering simple functions. Each model lists the number of neurons (variables) in each layer, and the activation function used in all the dense and convolutional layers is the Rectified Linear Unit (ReLU). The first layer of filters in OrbWeaver were fixed to log-transformed position weight matrices (see supplementary text). The Basset model was re-implemented using Theano and the Keras framework, and the batch-normalization steps were eliminated since they provided no increase in model accuracy for our problem at the cost of a large increase in model learning time.

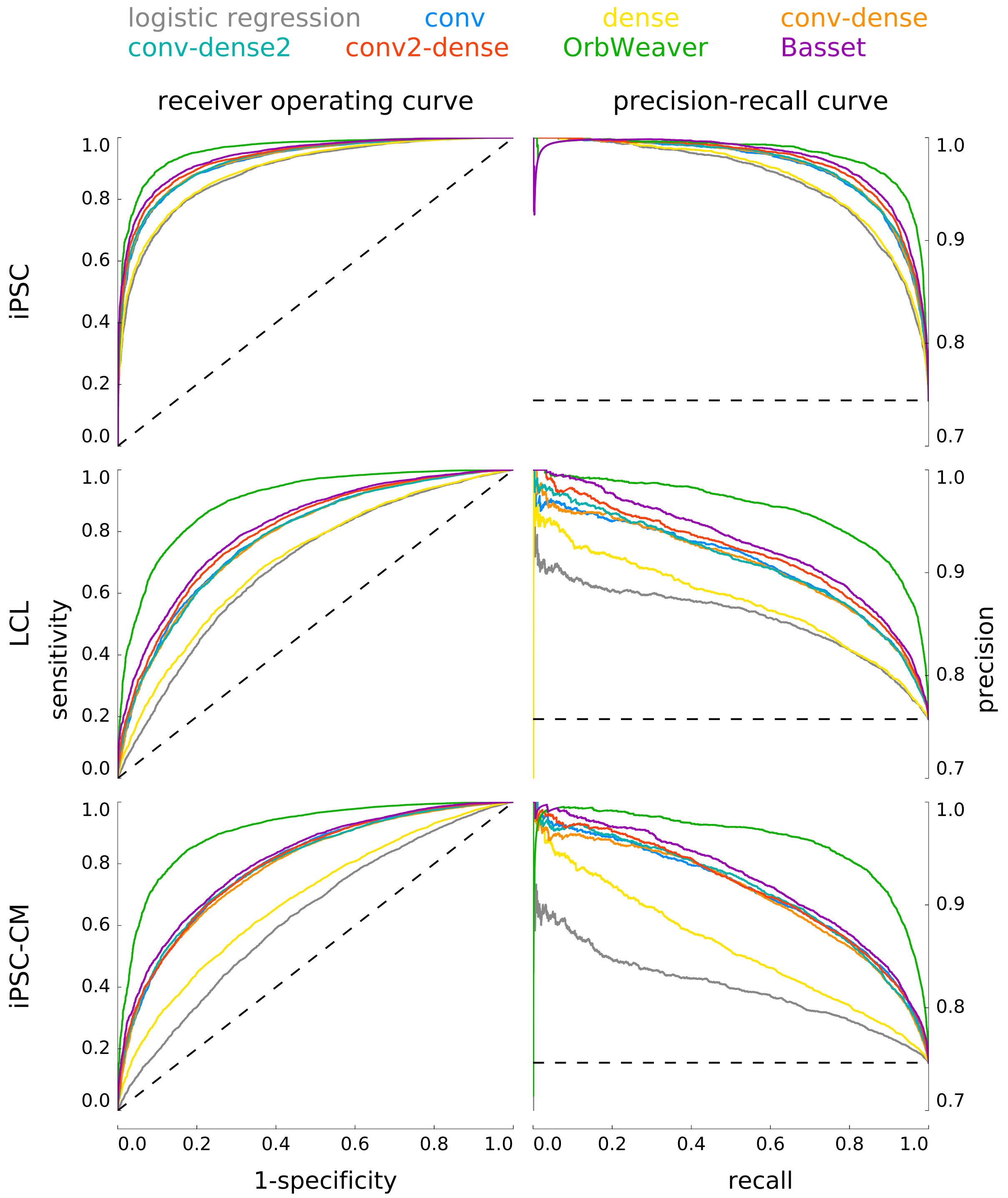

Figure S17: **Comparing the accuracy of neural network architectures.** The accuracies of each of the neural network models are compared in each cell type; the accuracies are represented using both the Receiver Operating Curve and the Precision-Recall curve. Notably, the gain in accuracy with each additional convolutional and dense layer was much smaller than the gain achieved by fixing the first convolutional filters to known position weight matrices of human transcription factors. In particular, OrbWeaver, a simpler neural network with known PWMs achieved a substantially higher accuracy than Basset, a more complex neural network.

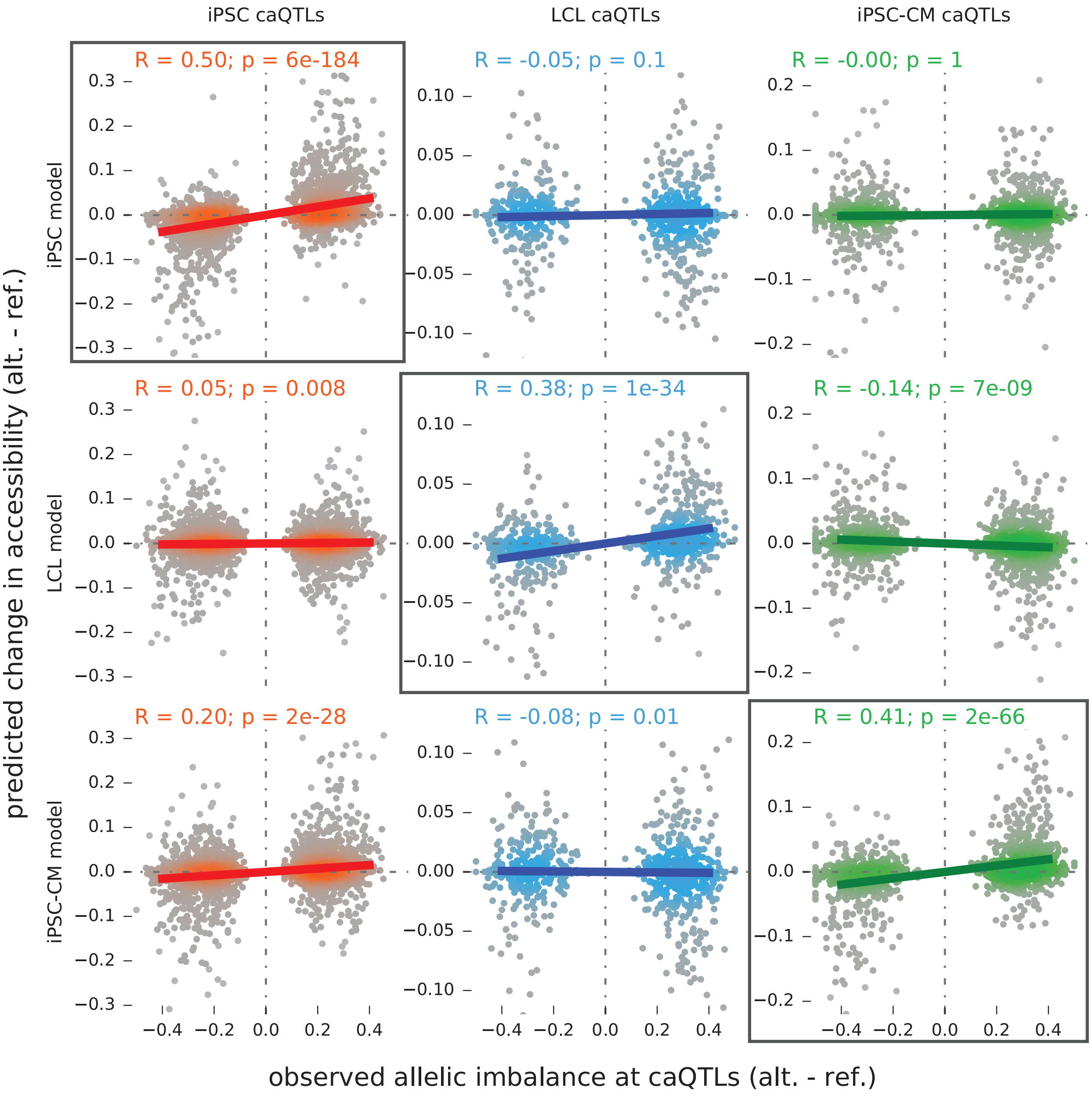

Figure S18: **Predicting genetic effects on chromatin activity using neural network models.** Observed allelic imbalance in ATAC-seq reads at caQTLs (estimated using WASP; x-axis) is compared with the difference in predicted chromatin activity between haplotypes tagged by the two alleles of the caQTL SNP (y-axis); both quantities are computed focusing on the causal caQTL SNP identified using qtlBHM, a Bayesian hierarchical model. Each column of panels corresponds to observed genetic effects in each of the three cell types (each point is a locus identified by qtlBHM as a caQTL with posterior greater than 0.99 in that cell type), and each row of panels corresponds to the genetic effects predicted by the OrbWeaver model relevant to each of the three cell types. Notably, in each cell type, predictions of genetic effect using a model for a different cell type has very low or no agreement with the observed allelic imbalance in chromatin activity (off-diagonal panels).

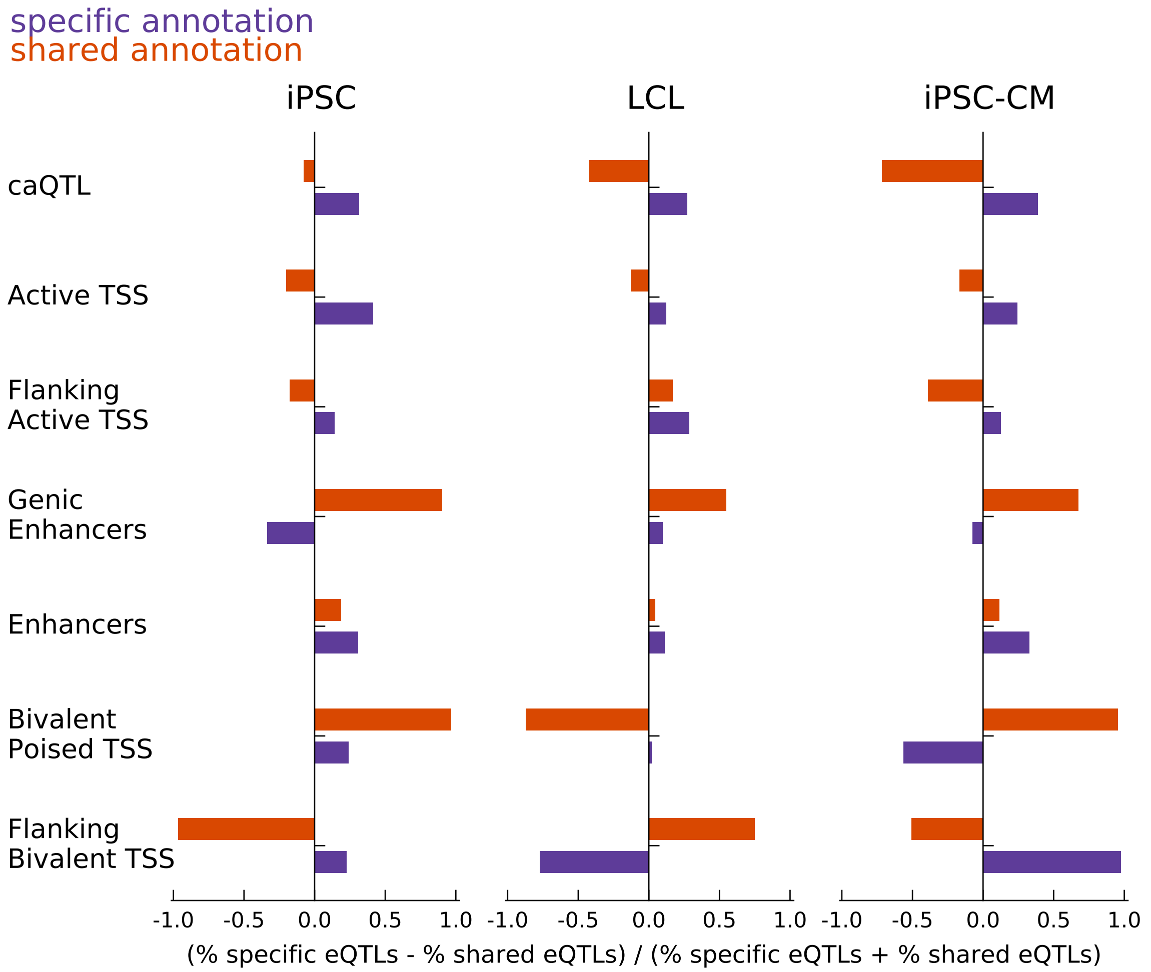

Figure S19: **Quantifying eQTLs explained by chromatin annotations.** For a set of genomic annotations that are specific to a cell type or shared between two or more cell types, we illustrate the difference in the proportion of shared eQTLs and the proportion of cell-specific eQTLs explained by each of the annotations. Importantly, we find that shared caQTLs and shared active TSS explain a larger fraction of shared eQTLs than cell-specific eQTLs. Enhancers, in general, explain a larger fraction of cell-specific eQTLs than shared eQTLs. In contrast, shared enhancers within genes explain a larger fraction of cell-specific eQTLs than shared eQTLs.
